# Prenatal Exposure to General Anesthesia Drug Esketamine Impaired Neurobehavior in Offspring

**DOI:** 10.1007/s10571-023-01354-4

**Published:** 2023-04-29

**Authors:** Ronghua Huang, Bingbiao Lin, Hongyan Tian, Qichen Luo, Yalan Li

**Affiliations:** 1 Department of Anesthesiology, The First Affiliated Hospital of Jinan University, Number 613, The West of Huangpu Avenue, Tianhe Region, Guangzhou, 510630 Guangdong Province China; 2 Department of Urology, Kidney and Urology Center, Pelvic Floor Disorders Center, The Seventh Affiliated Hospital of Sun Yat-Sen University, Shenzhen, 518000 Guangdong China

**Keywords:** Esketamine, Gestation, Neurobehavior, Offspring

## Abstract

Prenatal exposure to anesthetics has raised increasing attention about the neuronal development in offspring. Animal models are usually used for investigation. As a new drug, esketamine is the s-isoform of ketamine and is twice as potent as the racemic ketamine with less reported adverse effects. Esketamine is currently being used and become more favorable in clinical anesthesia work, including surgeries during pregnancy, yet the effect on the offspring is unknown. The present study aimed to elucidate the effects of gestational administration of esketamine on neuronal development in offspring, using a rat model. Gestational day 14.5 pregnant rats received intravenous injections of esketamine. The postnatal day 0 (P0) hippocampus was digested and cultured in vitro to display the neuronal growth morphology. On Day 4 the in vitro experiments revealed a shorter axon length and fewer dendrite branches in the esketamine group. The results from the EdU- imaging kit showed decreased proliferative capacity in the subventricular zone (SVZ) and dentate gyrus (DG) in both P0 and P30 offspring brains in the esketamine group. Moreover, neurogenesis, neuron maturity and spine density were impaired, resulting in attenuated long-term potentiation (LTP). Compromised hippocampal function accounted for the deficits in neuronal cognition, memory and emotion. The evidence obtained suggests that the neurobehavioral deficit due to prenatal exposure to esketamine may be related to the decrease phosphorylation of CREB and abnormalities in N-methyl-d-aspartic acid receptor subunits. Taken together, these results demonstrate the negative effect of prenatal esketamine exposure on neuronal development in offspring rats.

**Graphical Abstract:**

G14.5 esketamine administration influenced the neurobehavior of the offspring in adolescence. Poorer neuronal growth and reduced brain proliferative capacity in late gestation and juvenile pups resulted in impaired P30 neuronal plasticity and synaptic spines as well as abnormalities in NMDAR subunits. Attenuated LTP reflected compromised hippocampal function, as confirmed by behavioral tests of cognition, memory and emotions. This figure was completed on the website of Figdraw.

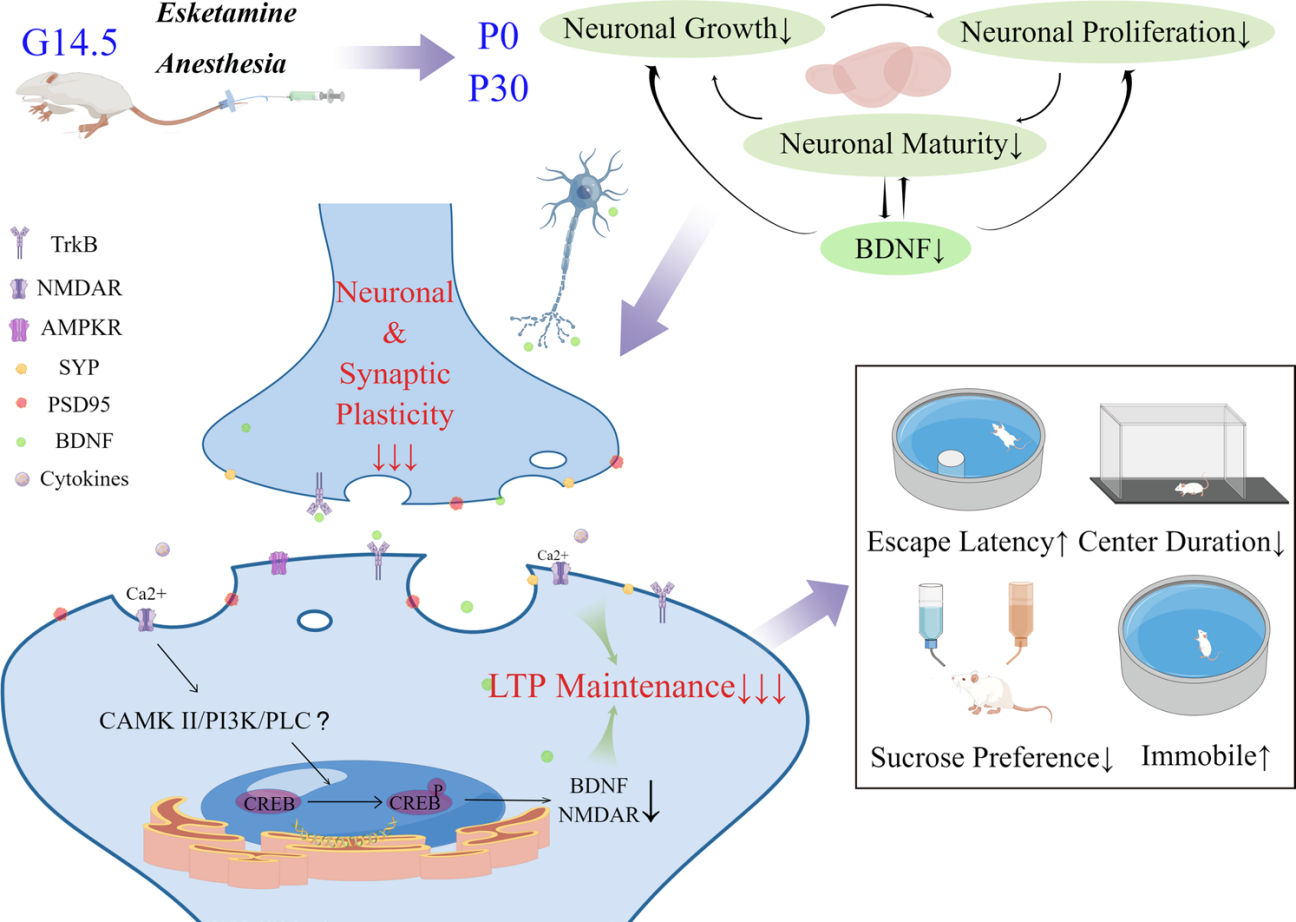

**Supplementary Information:**

The online version contains supplementary material available at 10.1007/s10571-023-01354-4.

## Introduction

It has been reported that nearly 2% of pregnant women undergo non-obstetric surgeries during pregnancy (Goodman 2002; Ni Mhuireachtaigh and O'Gorman 2006). In Denmark, the total incidence of surgery during pregnancy, including obstetric and non-obstetric surgery, is as high as 7% (Rasmussen et al. 2019). The US Food and Drug Administration (FDA) has called for more attention to perinatal anesthetic use (http://www.fda.gov/Drugs/DrugSafety/ucm532356.htm), although clinical human research is scarce. Ing et al. reported increased externalizing behavioral problems in childhood due to prenatal exposure to general anesthetics(Ing et al. 2021), which again aroused immediate concern for anesthesiologists, surgeons, and families(Fardelmann and Gaiser 2021).

Most of the research on the effects of prenatal exposure to anesthetics on offspring was based on animal models, such as the rat, mouse, rabbit (Van der Veeken et al. 2019), and rhesus macaque (Brambrink et al. 2012). Different anesthetics may exhibit different effects, either positive or negative. Prenatal exposure to sevoflurane was reported to inhibit fetal neural stem cell proliferation (Wang et al. 2018), neuronal migration and axon growth (Chai et al. 2019, 2020), cause neuronal apoptosis(Zhang et al. 2017; Zheng et al. 2013) and a persistent imbalance of excitatory and inhibitory neurons in the medial prefrontal cortex (Zhao et al. 2020), leading to learning and memory deficits and anxiety-like behaviors. In contrast, dexmedetomidine could reverse sevoflurane-induced toxicity (Shan et al. 2018). Similar negative effects of traditional ketamine were found as that of sevoflurane.

There are two optical isomers of the 2-(2-chlorophenyl)-2-(methylamino)-cyclohexanone ketamine: S(+) ketamine and R(−) ketamine(Morgan et al. 2012). Ketamine was first applied to patients requiring surgery as a pretreatment or for sedation, and also for induction and maintenance of general anesthesia (Curran and Morgan 2000; Domino et al. 1965). In addition, a subanesthetic dose of ketamine is used for acute and chronic pain relief in anesthesiology, emergency, and cancer internal medicine departments (Allen and Ivester 2018; Brinck et al. 2018; Gao et al. 2016; Mercadante et al. 2000; Vadivelu et al. 2016). In addition to the anesthetic and analgesic effect of ketamine, the S-isomer, esketamine was found to have an antidepressive effect, especially for patients with treatment-resistant depression (Canuso et al. 2018; Daly et al. 2018; Shouan and Grover 2018; Singh et al. 2016). Esketamine was approved to treat treatment-resistant depression in 2019 by the US FDA (Cristea and Naudet 2019), and was also approved by the Chinese National Medical Products Administration in 2020. Ketamine is water- and lipid-soluble, so it can be easily administered via various routes, such as intravenous, intramuscular, intranasal, oral, and rectal (Gao et al. 2016). Once unpopular due to its dissociative side effects, ketamine is now back to its former glory thanks to the numerous medical effects it exerts through multiple pathways (Nowacka and Borczyk 2019).

Although ketamine is commonly used in clinical anesthesia work for surgeries during pregnancy, anesthesiologists prefer to use esketamine after its approval for clinical use, as it has less adverse effects and a shorter recovery time than ketamine (Wang et al. 2019). Esketamine has a higher affinity for the N-methyl-d-aspartic acid receptor (NMDAR) than ketamine, which may exert different effects on the offspring. Nevertheless, the effect of perinatal use of esketamine on the fetus and offspring is unknown.

In the current study, we aimed to clarify the effect of prenatal exposure to esketamine on offspring using a pregnant rat model. Neuronal development, including neuronal growth, proliferation and survival, as well as synaptogenesis were evaluated by morphological experiments. Electrophysiological and neurobehavioral tests were performed to assess brain function.

## Materials and Methods

### Subjects

All experimental procedures were performed according to the guidelines that have been approved by the Ethics Committees of Jinan University, Guangdong, China (Approval number: IACUC-20201215-05). Animals were housed in cages in a temperature- and humidity-regulated room with a 12-h light/dark cycle and were allowed free access to water and food. All efforts were made to minimize the number of animals used. A total of 8 female and 4 male 8-week-old rats per group were used. One male and two female rats were housed in one cage for mating at the beginning. After vaginal plugs were detected in the morning, pregnant female rats were housed alone until delivery (1 pregnant rat in a cage). This day was marked as gestational Day 0.5 (G0.5).

**Fig. 1:**
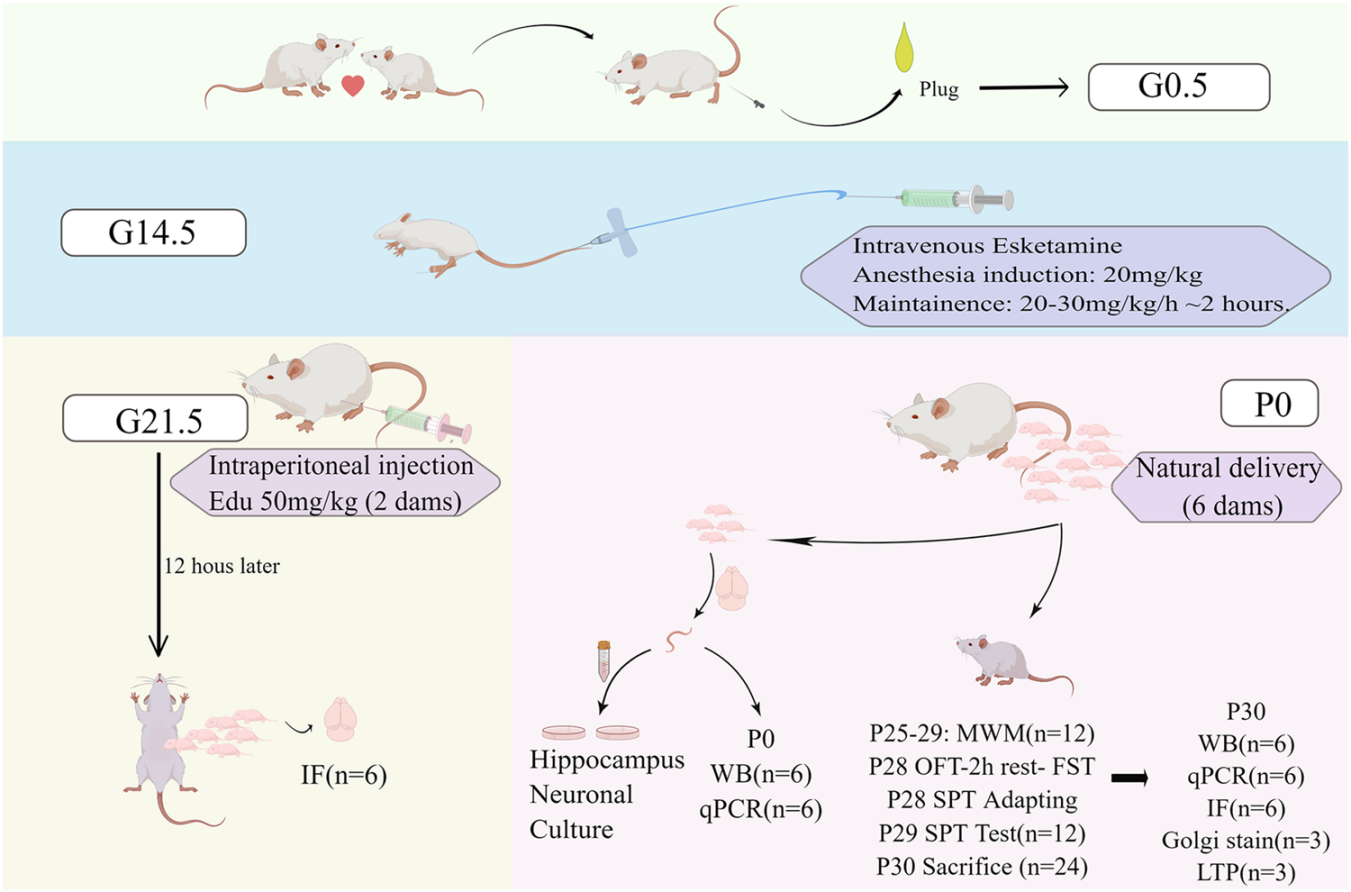
Flowchart of the experiment protocols. Gestational Day 14.5 (G14.5) dams (n = 8/group) were randomly divided into the esketamine group or the control group and received injection of either esketamine or normal saline. At G21.5, 2 dams/group received EdU injection and cesarean section 12 h later. The newborn pups were sacrificed for brain collection. The other 6 dams were allowed to give birth naturally. At postnatal Day 0(P0), 4 pups from each group were sacrificed for hippocampal neuron culture. In addition, hippocampal RNA and protein were extracted at P0, respectively. The other pups were allowed to grow up and take the behavioral test. At P30, adolescent pups were sacrificed for different sample extractions or LTP recordings. *G* gestational day, *P* postnatal day, *EdU* 5-ethynyl-2’-deoxyuridine, *MWM* Morris water maze test, *OFT* open field test, *FST* forced swimming test, *SPT* sucrose preference test, *WB* western blot, *qPCR* quantitative real-time PCR, *IF* immunofluorescence, *LTP* long-term potentiation. This figure is completed on the website of Figdraw

Dams were used for experiments on gestational Day 14.5 (G14.5) and were divided randomly into the esketamine group and the control group. Rats in the esketamine group were given a bolus dose of 20 mg/kg esketamine via the tail vein and then maintained with 20–30 mg/kg/h esketamine for 2 h in a sedative state between light anesthesia and deep sedation, evidenced by a lack of voluntary movement, decreased muscle tone, and minimal reaction to painful stimulation. The body temperature of the dams was maintained between 36.5 and 37.5 °C by a temperature-settled heating pad throughout the experiments. After recovery from the 2-h continuous intravenous infusion anesthesia, the rats were returned to their cages. On the contrary, dams in the control group received the intravenous injection of the same induction volume of normal saline and were then sent back (Fig. 1).

Two dams per group were injected with the 50 mg/kg 5-Ethynyl-2ʹ-deoxyuridine (EdU) (APExBIO, Houston, USA, B8337) intraperitoneally at gestational Day 21.5 and were anesthetized with sevoflurane 12 h later, receiving a cesarean section if they had not delivered. Newborn postnatal Day 0 (P0) pup brains were collected for immunohistological examination of the EdU-positive test. The remaining dams were allowed to give birth naturally, and the day pups were delivered was recorded as P0. Four P0 pups per group were used for hippocampal neuron culture. A total of 6 pups (3 pups per dam and 2 dams per group, n = 3 × 2 = 6) were sacrificed for the extraction of protein and 6 for the extraction of RNA from the bilateral hippocampi on P0. The other pups were left to grow up under the breastfeeding of their mothers (no more than 6 pups per cage were kept with their mother) and were weaned on P21. Two to three offspring rats were housed in one cage from P21-P30. Behavioral tests were carried out on P24-29. At the same time, 6 pups per group received intraperitoneal injections of 50 mg/kg EdU daily on P27-29, and their brains were collected on P30 after euthanasia. Besides, at P30, 6 pups per group were sacrificed for extraction of protein and RNA respectively, and 3 pups per group were sacrificed for the Golgi staining test, while 3 were sacrificed for long-term potential (LTP) evaluation of the brain slices.

### Behavioral Test

Twelve pups per group were randomly selected for the Morris water maze (MWM) test on P24-29. Another 12 pups per group were randomly selected for the open field activity test (OFT) on P28 and the forced swimming test (FST) at least after a three-hour rest. The sucrose preference test (SPT) was performed on P28-29. To avoid possible behavioral biases, each behavioral test of the two groups of animals was performed at the same time interval on the day of testing.

#### Morris Water Maze (MWM)

The MWM test was performed to assess spatial learning and memory in the animals. Rats were allowed to swim in a water tank 150 cm in diameter and 100 cm in height with a depth of 80 cm water, where an escape platform (15 cm in diameter) was placed 1 cm below the water surface. The water temperature was maintained at 25 ± 1 °C and the rats were placed in the pool in one quadrant and allowed to swim for 1 min to search for the platform for four trials per day (beginning from the four quadrants). On the training day of P24-28, if the rat had not arrived at the platform in one minute, it was guided to the platform and left there for 30 s, and the escape time was recorded as 60 s. On P29, the platform was withdrawn, and the rats were allowed to swim for one minute. Data were recorded with a video camera and analyzed with EthoVision XT 7.0 (Noldus).

#### Open Field Activity Test (OFT)

The open field activity test was applied to measure the general locomotor activity and anxiety-like behaviors of the rats (Prut and Belzung 2003). On P28, pups were left in an apparatus (100 cm × 100 cm) with walls and a black background and were allowed to move freely for 10 min. The arena was cleaned with 75% ethanol after each trial. Data were recorded with a video camera and analyzed by EthoVision XT 7.0 (Noldus). The duration of the center zone traveling was used to measure anxiety-like behaviors, and the total traveling distance and speed were used to measure locomotor activity.

#### Forced Swimming Test (FST)

The FST is regarded as a behavioral test of the depressive extent (Petit-Demouliere et al. 2005). After a three-hour rest period from the open field test, pups were placed in a transparent cylinder (50 cm high and 15 cm in diameter) with a depth of 25 cm water which was maintained at 25 ± 1 °C. Pups were left to swim for 2 min to adapt to the environment and then another 5 min to swim, being recorded by a video camera connecting the EthoVision XT 7.0 software (Noldus). The immobile time of the pups was calculated. A variance of the movement of less than 20% was defined as immobile.

#### Sucrose Preference Test (SPT)

It is acknowledged that rodents prefer sweet food and beverages when they have a choice between regular water and a sucrose solution (Goshen et al. 2008; Zhou et al. 2011). The ratio of sucrose consumption to the total intake is regarded as the taste preference, and generally speaking, less sucrose consumption reveals more severe anhedonia (Rygula et al. 2005). At P28, pups were allowed alone in a cage with 2 bottles, one containing 1% (wt/vol.) sucrose in double distilled water and one with double distilled water alone. They were left to adapt to the environment of the new cage and bottles until the next day. At P29, they were deprived of both water and food for 8 h. Then, they were given one bottle of regular water and one bottle of sucrose in an exchange position for 1 hour. The pre- and post-weight of the two bottles were recorded, so that the consumption could be measured. Sucrose preference was quantified as the ratio of sucrose intake to total fluid intake.

### Brain Slice Section Preparation

At P0 and P30, pups received transcardial perfusion with PBS and then 4% paraformaldehyde (4%PFA). The whole brain was separated from the cranium. The brain was postfixed with 4% PFA overnight and then dehydrated in 10%, 20%, and 30% sucrose. The P0 brain was cut with a cryostat microtome (Thermo) into 14 µm/slice, while the P30 brains were cut with a sliding microtome (Leica) into 40 µm/slice.

### Immunofluorescence

Brain sections were blocked with 3% bovine serum albumin and 10% donkey serum in 0.3% PBST (1X PBS with 0.3% Triton-100) for 2 h. Then, the slices were incubated overnight with the primary antibody at 4 °C and washed for 10-min ~ 3 times with 0.3% PBST, followed by a 2-h incubation with the secondary antibody at room temperature. The primary antibodies used in this study included anti-NeuN (1:1000, Abcam, 104224), anti-DCX (1:1000, Abcam, 18723), and anti-beta III tubulin (1:1000, Abcam, 18207). Secondary antibodies used were as follows, donkey anti-mouse 488 IgG (1:1000; Thermo Fisher, USA, A21202), and donkey anti-rabbit 546 IgG (1:1000; Thermo Fisher, USA, A10040). DAPI (1:1000; Cell Signaling Technology, 4083) was used for staining the nucleus. The EdU-positive test was accomplished with the help of an EdU imaging kit (APExBIO, Houston, USA, K1076) according to the manufacturer’s protocol. Images were captured via a Zeiss LSM700 confocal microscope and Zeiss software.

### Western Blot

Proteins were extracted using RIPA lysis buffer (Beyotime, P0013B) with protease and phosphatase inhibitor cocktail (Beyotime, P1045) on ice, homogenized via ultrasonication and centrifuged at 12,000×*g* for 15 min at 4 °C. The supernatant was assessed using a BCA assay kit (Beyotime, P0012) and then quantified to the same concentration with RIPA lysis buffer and loading buffer (Beyotime, P0015) and boiled at 100 °C for 10 min. Samples were then separated on an 8 to 12% SDS-PAGE gel (Beyotime, P0012A) and transferred to polyvinylidene fluoride (PVDF) membranes (Merck Millipore). Membranes were blocked with 5% BSA in 0.1% TBST (TBS with 0.1% Tween 20) for two hours at room temperature and then incubated with the primary antibodies overnight at 4 °C. After three 10-min washes in 0.1% TBST, the membranes were incubated with secondary antibodies for 2 h at room temperature and then washed 3 times. The blots were visualized with a ChemiDoc Touch Imaging System (Bio-Rad) and quantified using ImageJ.exe. The primary antibodies were: anti-BDNF (1:1000, Abcam, ab108319), anti-SY38 (1:500, Abcam, ab8049), anti-PSD95 (1:1000, CST, 3450), anti-p-CREB (1:500, CST, 9198), anti-CREB (1:1000, PTM, 5595), anti-NR1 (1:1000, Millipore, 05-432), anti-NR2A (1:1000, Millipore, 07-632), anti-NR2B (1:1000, Millipore, 06-600), anti-β-tubulin (CST, 2146). The secondary antibodies were HRP goat anti-rabbit (1:5000, Abcam, ab6721) and HRP goat anti-mouse (1:5000, Abcam, ab6789).

### qPCR

Total RNA was extracted using TRIzol (Invitrogen, Carlsbad, CA) reagent following the manufacturer’s guidance and then assessed by Nanodrop 2000 (Nanodrop, USA). One microgram of RNA was reverse transcribed into cDNA using HiScript II Q RT SuperMix for qPCR (Vazyme, R223-01, China). qPCR were performed with TB green Premix Ex Taq II (Takara, RR820A, Japan) using a Bio-Rad CFX96 machine. The primers are listed in Table 1.

**Table 1:** qPCR primers used in this study

		Primer
BDNF	Forward	TCCATTCAGCACAAGGGTCC
	Reverse	CACTAACACATTCGCGCTGG
Dlg4	Forward	ACTACTCCTCGTCGGCTGAA
	Reverse	GGCTGTAGCCAGAAAGTCCATC
SYP	Forward	TCCTGTACCCTCTGCTGTGT
	Reverse	GCACAGGAAAGTAGGGGGTC

### Golgi Staining

P30 rat brains were processed and stained using the FD rapid Golgi Stain Kit (FD Neurotechnologies, Inc, PK401). According to the manufacturer’s protocol, well-stained individual neurons in the CA1 or CA3 area were selected for three-dimensional reconstruction, and dendrite analysis was performed with the help of image J. Images of ten segments of dendrites per animal were acquired with a 63X objective Zeiss microscope.

### Cell Culture

The Hippocampi of the P0 pups were dissociated with 0.125% trypsin, and the reactions were stopped by fetal bovine serum (FBS). After centrifugation at 1000 rpm for 3 min, the samples were suspended in DMEM F12 and seeded on 6-well plates precoated with poly-l-lysine hydrobromide (Solarbio, P8130). Neurons were cultured with 10% FBS in DMEM F12 for the first 8 h, and then the culture medium was changed to Neurobasal with 2% B27. Half of the culture medium was changed 48 h later. On Day 4 in vitro, plates were fixed with 10% PFA, followed by immunofluorescence staining for βIII tubulin.

### Electrophysiology

Hippocampal slices (300 µm) were prepared from the P30 pup brains, immediately incubated at 32 °C for 30 min and maintained at 26 °C for 1 h as described previously (Toni et al. 1999). The slices were then placed in a recording chamber at 25 °C and perfused with oxygenated artificial cerebrospinal fluid (ACSF) containing 126 mM NaCl, 2.5 mM KCl, 1.25 mM NaH_2_PO_4_, 26 mM NaHCO_3_, 10 mM glucose, 2 mM CaCl_2_, and 2 mM MgSO_4_ at a rate of 1 ml/min. A glass electrode filled with ACSF (2–3 MΩ) was placed in the CA1 area to record the field excitatory postsynaptic potential(fEPSP). The Schaffer collateral pathway was stimulated every 30 s using concentric bipolar electrodes. The theta burst stimulation (TBS) protocol (four pulses of 100 Hz repeated three times at 5 Hz, and a 20 s intertrain interval) was used to induce LTP. Field potentials were amplified, lowpass filtered (MultiClamp 700B, Axon Instruments), and digitized, and the data were analyzed using Clampex software (Axon Instruments).

### Statistical Analysis

All data are presented as the mean ± SEM. Homogeneity of variance was verified using Levene's test, and then a single comparison between both groups was made using an unpaired two-tailed Student’s t test with the help of GraphPad Prism software 8.0.1. P < 0.05 was considered significant.

## Results

1. Gestational administration of esketamine impaired the growth status of the P0 cultured neurons.

**Fig. 2:**
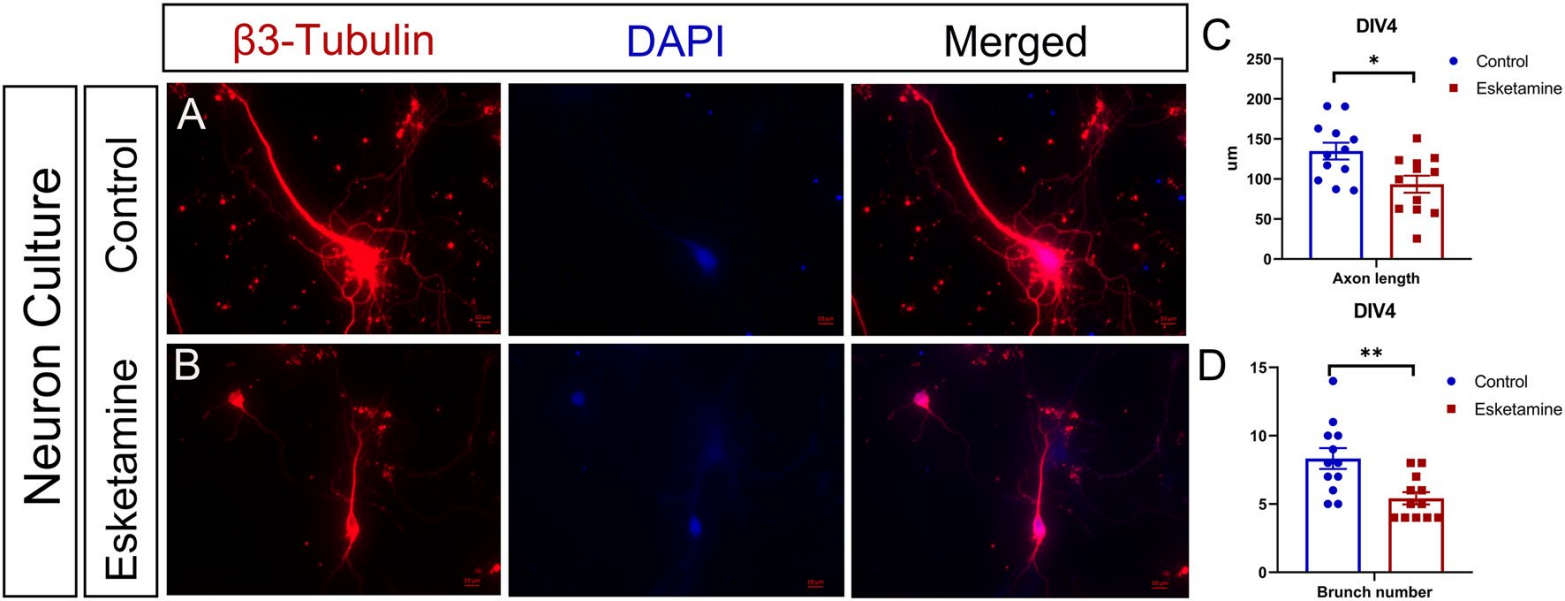
Morphology of the neurons on Day 4 in vitro. The P0 hippocampus was digested and seeded in the medium for neuron culture. Four pups from 2 different dams were used, and the hippocampal neurons were seeded in 3 wells for each pup (n = 12). Thus, there were 12 wells in each group. On Day 4 in vitro, cultured neurons were fixed with PFA and then incubated with beta III tubulin primary antibody overnight. Three neurons from every well were used for analysis. The axons of the neurons from the esketamine group were significantly shorter than those from the control group (t test, 134.8 ± 10.5 µm vs. 93.5 ± 10.6 µm, P = 0.011). Moreover, the number of the dendrite branches was much smaller (t test, 5.4 ± 0.5 vs. 8.3 ± 0.8, P = 0.003). Data are shown as the mean ± SEM. *P < 0.05, and **P < 0.01. Scale bar: 10 µm

We used a rat model to investigate the effect of general anesthesia during pregnancy on the offspring. On gestational day 14.5, pregnant rats were injected with esketamine via the tail vein for anesthesia induction and maintenance. To study the growth status of postnatal neurons after delivery, we cultured neurons from the P0 hippocampus. On Day 4 of the in vitro experiment, the cultured neurons were fixed with PFA and then cultivated with beta III tubulin primary antibody overnight to visualize the neuronal cytoskeleton. The imaging (Fig. 2) revealed that the axons of the neurons from the esketamine group were significantly shorter than those from the control group (t test, 134.8 ± 10.5 µm vs. 93.5 ± 10.6 µm, P = 0.011). Moreover, the number of dendrite branches was much smaller (t test, 5.4 ± 0.5 vs. 8.3 ± 0.8, P = 0.003). Hippocampal neurons cultured from the esketamine group pups seem to be less robust in axonal growth and dendrite branches number increase.

(2) Gestational administration of esketamine impaired the proliferative capacity of the offspring’s brain.

**Fig. 3:**
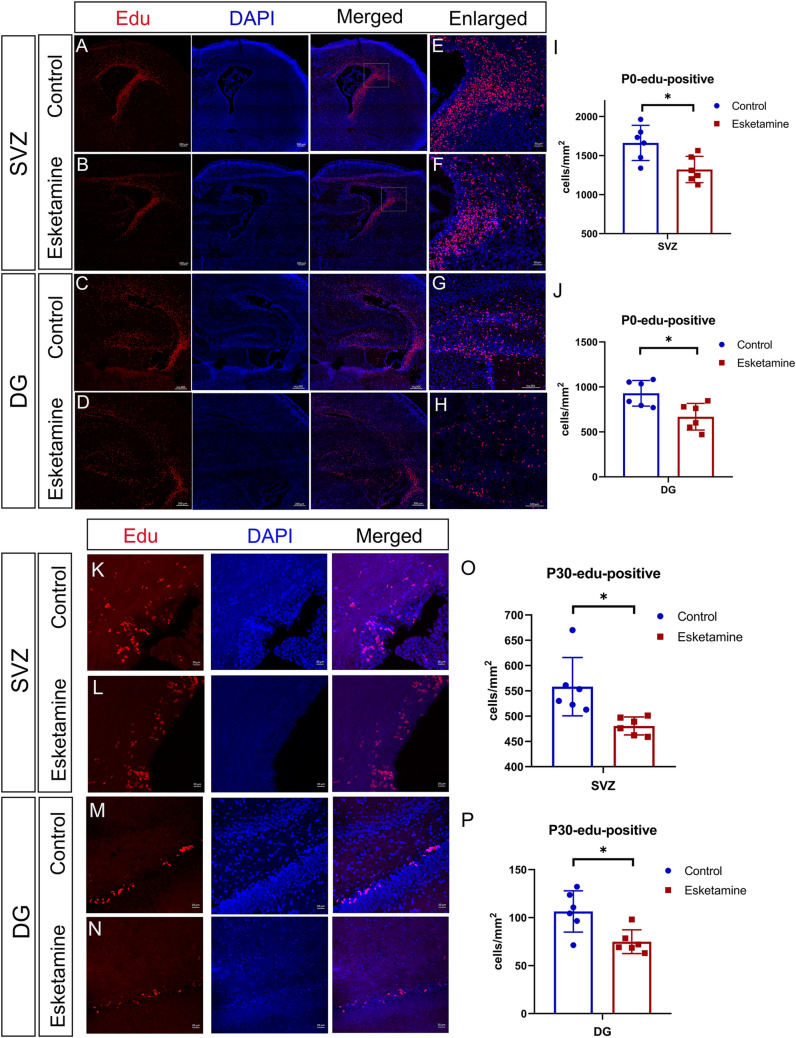
EdU-positive cells in the SVZ and DG, both in P0 and P30. G21.5 pregnant rats (n = 2) received an intraperitoneal injection of 50 mg/kg EdU once and received a cesarean section 12 h later. Three P0 brains from each dam (n = 6) were collected for the EdU-positive test. In addition, pups received intraperitoneal injections of 50 mg/kg/d EdU on P27-29 and were sacrificed on P30 for brain collection (n = 6). **A**–**H** are representative images of the P0 EdU-positive results, of which **E**–**H** are the enlarged views. **I**–**J** were quantitative analyses of P0 EdU-positive cells in the SVZ and DG, respectively. Significant differences were found between the two groups (t test, Esketamine vs Control: SVZ 1321 ± 69 vs. 1661 ± 92 cells/mm^2^, P = 0.014; DG 668 ± 60 vs. 929 ± 58 cells/mm^2^, P = 0.011). K-N are representative images of the P30 EdU-positive results, and quantitative (**O**, **P**) analysis showed remarkable differences (t test, Esketamine vs. Control: SVZ 481 ± 7 vs 558 ± 24 cells/mm^2^, P = 0.010; DG 75 ± 5 vs. 106 ± 9 cells/mm^2^, P = 0.011). Data are shown as the mean ± SEM. *P < 0.05. Scale bar: **A**–**D**: 200 µm; **E**–**H**: 100 µm; **K**–**N**: 20 µm

As the subventricular zone(SVZ) and the dentate gyrus(DG) of the hippocampal formation are acknowledged to produce new neurons (von Bohlen und Halbach 2011), we focused on the exploration of EdU-positive neurons in these two areas. As shown in Fig. 3, the number of EdU-positive neurons in the SVZ and DG in the esketamine group was significantly reduced compared to that in the control group in P0, suggesting an impaired proliferative potential caused by esketamine from midgestation to the end of pregnancy. Moreover, P30 EdU-positive images showed similar results that the esketamine group had significantly fewer EdU-positive cells in both the SVZ and DG than the control group. The above results indicated that the impaired proliferation of the brain may last until adolescence in rats.

(3) Gestational administration of esketamine influenced the neuronal plasticity of the offspring brain in adolescent rats.

**Fig. 4:**
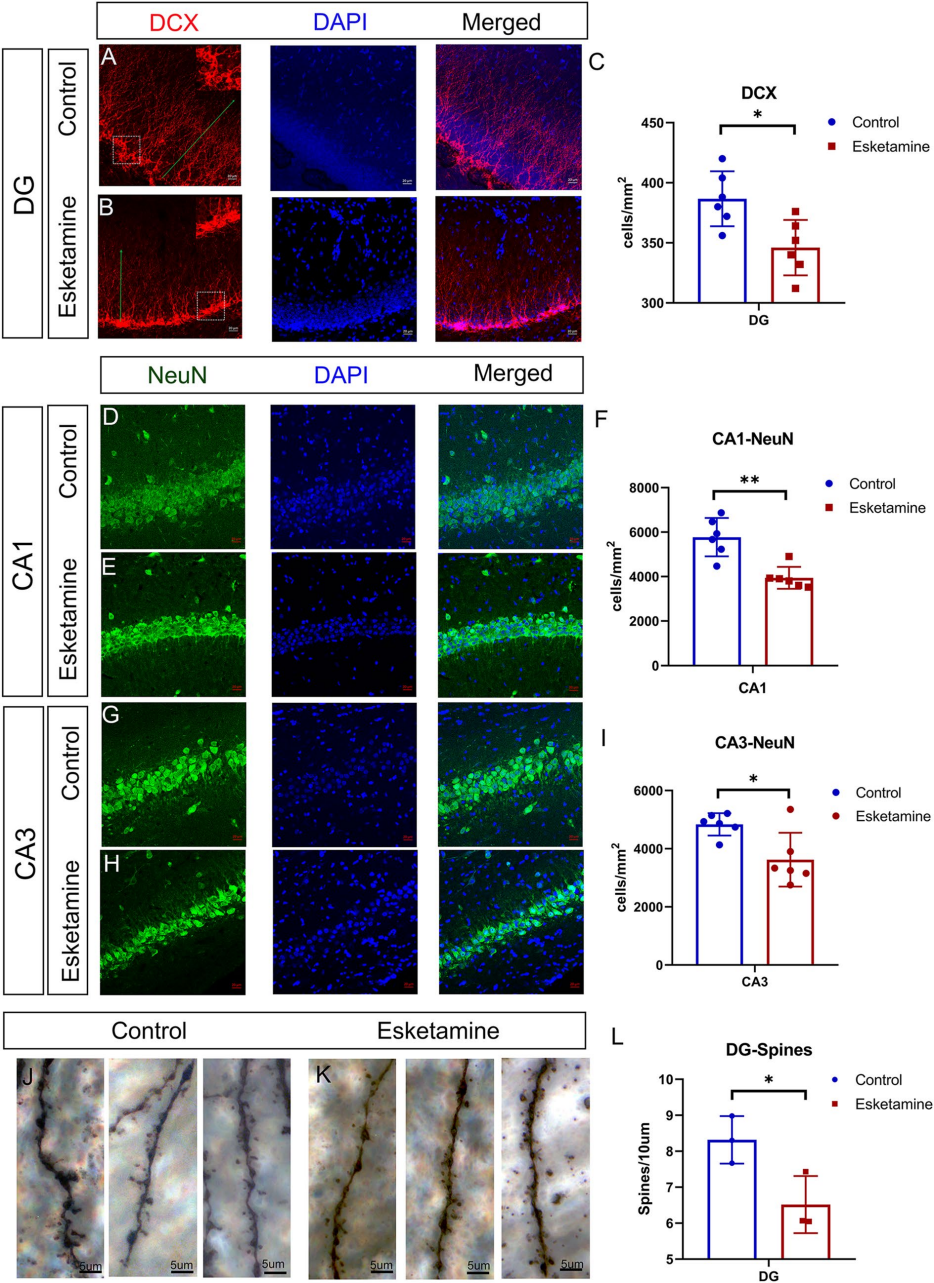
Morphology of the P30 brain slices. Naturally delivered pups grown to P30 were sacrificed for brain slice collection. **A**, **B** are images of the immunofluorescence staining for DCX (newly emerging neuron marker) in the dentate gyrus. Quantitative analysis (**C**) showed a significantly reduced number of DCX-positive cells in the esketamine group (n = 6) (t test, Esketamine vs. Control 346 ± 9 vs. 387 ± 9 cells/mm^2^, P = 0.012). Additionally, the morphology showed longer and more branches (green arrow) in the control group (without quantification). **D**, **E** and **G**, **H** are images of the immunofluorescence staining for NeuN (a mature neuron marker) in CA1 and CA3 regions, respectively. **F**–**I** show a significant reduction in NeuN-positive cells in the esketamine group, both in CA1 and CA3 (n = 6) (t test, Esketamine vs. Control CA1 3943 ± 203 vs. 5773 ± 352 cells/mm^2^, P = 0.001; CA3 3622 ± 377 vs. 4837 ± 158 cells/mm^2^, p = 0.014). **J**–**K** are images of the Golgi-stained brain slices, while **L** is the quantitative result of the number of dendritic spines in every 10 µm branches, with a significant difference (n = 3) (t test, Esketamine vs. Control 6.5 ± 0.5 vs. 8.3 ± 0.4, P = 0.039). Data are shown as the mean ± SEM. *P < 0.05. Scale bar: **A**, **B**, **D**, **E**, **G**, **H**: 20 µm; **J**, **K**: 5 µm

Neuronal plasticity is reflected by the addition of new and functional neurons as well as dendritic spines (von Bohlen Und Halbach and von Bohlen Und Halbach 2018). To display the neurogenesis and maturity of the brain in adolescent rats directly, we performed immunofluorescence tests of DCX and NeuN as well as Golgi staining in P30 brain slices. DCX marks the newly emerging neurons in the hippocampal dentate gyrus (Brown et al. 2003), reflecting neurogenesis. Figure 4A–C shows that the number of DCX-positive cells in the dentate gyrus was significantly reduced in the esketamine group (t test, 346 ± 9 vs. 387 ± 9 cells/mm^2^, P = 0.012). Besides, NeuN is a marker of mature neurons (Duan et al. 2016), and Golgi staining is one of the most effective techniques for studying the morphology of neuronal dendrites and dendritic spines(Du 2019). We found that the number of mature neurons in the esketamine group could not reach the corresponding level in the control group, either in the CA1 or in the CA3 region (t test, CA1 3943 ± 203 vs. 5773 ± 352 cells/mm^2^, P = 0.001; CA3 3622 ± 377 vs. 4837 ± 158 cells/mm^2^, P = 0.014) (Fig. 4D–I). In addition, the spine density (Fig. 4J–L) decreased significantly in the esketamine group (t test, 6.5 ± 0.5 vs. 8.3 ± 0.4 /10 µm, P = 0.039). Morphological studies of the P30 hippocampus revealed impaired neuronal and synaptic plasticity.

(4) Mechanism of the impaired neuronal development.

**Fig. 5:**
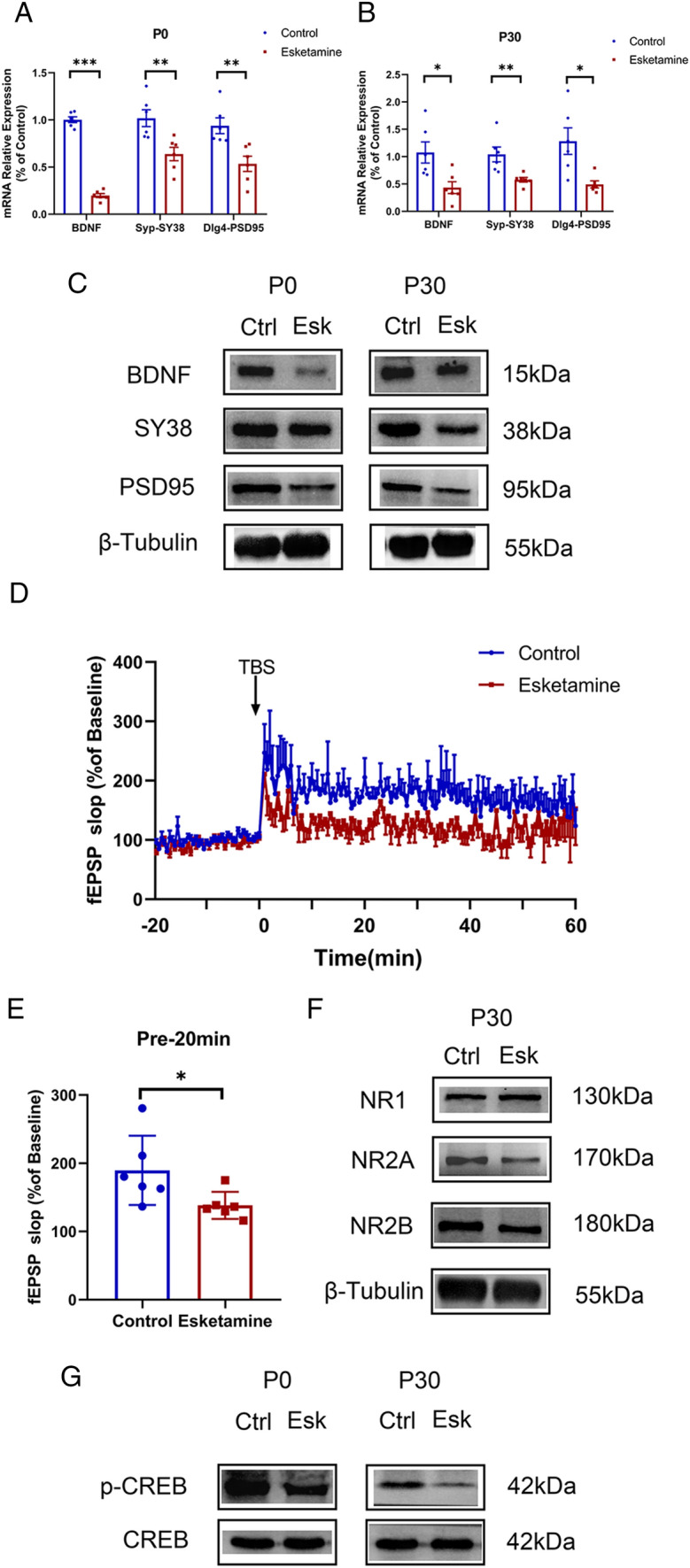
Mechanism of the impaired neuronal development. P0 and P30 naturally delivered pups were sacrificed for extraction of hippocampal RNA and protein (n = 6 pups/group respectively). **A**–**C** Show that the transcriptional and translational levels of BDNF, SY38 and PSD-95 all decreased significantly, in both the P0 and P30 hippocampus. LTP was attenuated in the pups from the esketamine group and the normalized fEPSP slope (last 20 min) decreased significantly (n = 6, 3 pups/group and 2 brain slices per pup used for recording) (t test, 138.3 ± 8.1 vs. 189.6 ± 20.8, P = 0.044) (**D**, **E**). **F** shows that there was no difference in NR1 levels between the two groups, whereas NR2A and NR2B expression was significantly reduced in the esketamine group in the P30 hippocampus. In addition, the phosphorylation of CREB was weakened in the esketamine group, at both P0 and P30 (**G**). *Ctrl* control group, *Esk* esketamine group

The hippocampus is composed of the cornu ammonis (areas CA1–CA3), the dentate gyrus (DG) and the subicular complex (Squire and Zola 1996). Impaired neuronal plasticity may indicate weakened hippocampal function and synaptogenesis. As brain-derived neurotrophic factor (BDNF) is a key element in hippocampal function (Leal et al. 2017; von Bohlen Und Halbach & von Bohlen Und Halbach, 2018), and synaptogenesis relies on synaptic proteins (Petzoldt and Sigrist 2014), we detected the relative mRNA and protein levels of BDNF, synaptophysin (SY38) and postsynaptic density 95 (PSD95). Figure 5A–C shows that the mRNA and protein levels of BDNF, SY38 and PSD-95 decreased significantly in both the P0 and P30 hippocampus.

Considering that the increased spine densities and formation of new and mature functional synapses play a crucial role in Long-term potentiation (LTP) (Toni et al. 1999), we performed the LTP test on P30 brain slices of the pups. The results showed that LTP was attenuated in the hippocampus of the pups from the esketamine group. The normalized fEPSP slope (last 20 min) (Chen et al. 2022) for hippocampal slices decreased significantly (t test, 138.3 ± 8.1 vs. 189.6 ± 20.8, P = 0.044) (Fig. 5D–E).

In addition, because synaptic development could be modulated by NMDA receptors (Pagano et al. 2021), we detected the P30 hippocampal NR1, NR2A, and NR2B subunits using western blotting (Fig. 5F). The results showed that the NR1 level did not differ between the two groups. However, NR2A and NR2B expression decreased significantly in the esketamine group in the P30 hippocampus. The CREB signaling pathway was reported to enhance neurogenesis and improve cognitive function (Cohen and Greenberg 2008; Lonze and Ginty 2002). We found that phosphorylated CREB, the active functional form, was expressed at lower levels in the hippocampus at both P0 and P30 in the esketamine group.

(5) Impaired neuronal development induced behavioral deficiency.

**Fig. 6:**
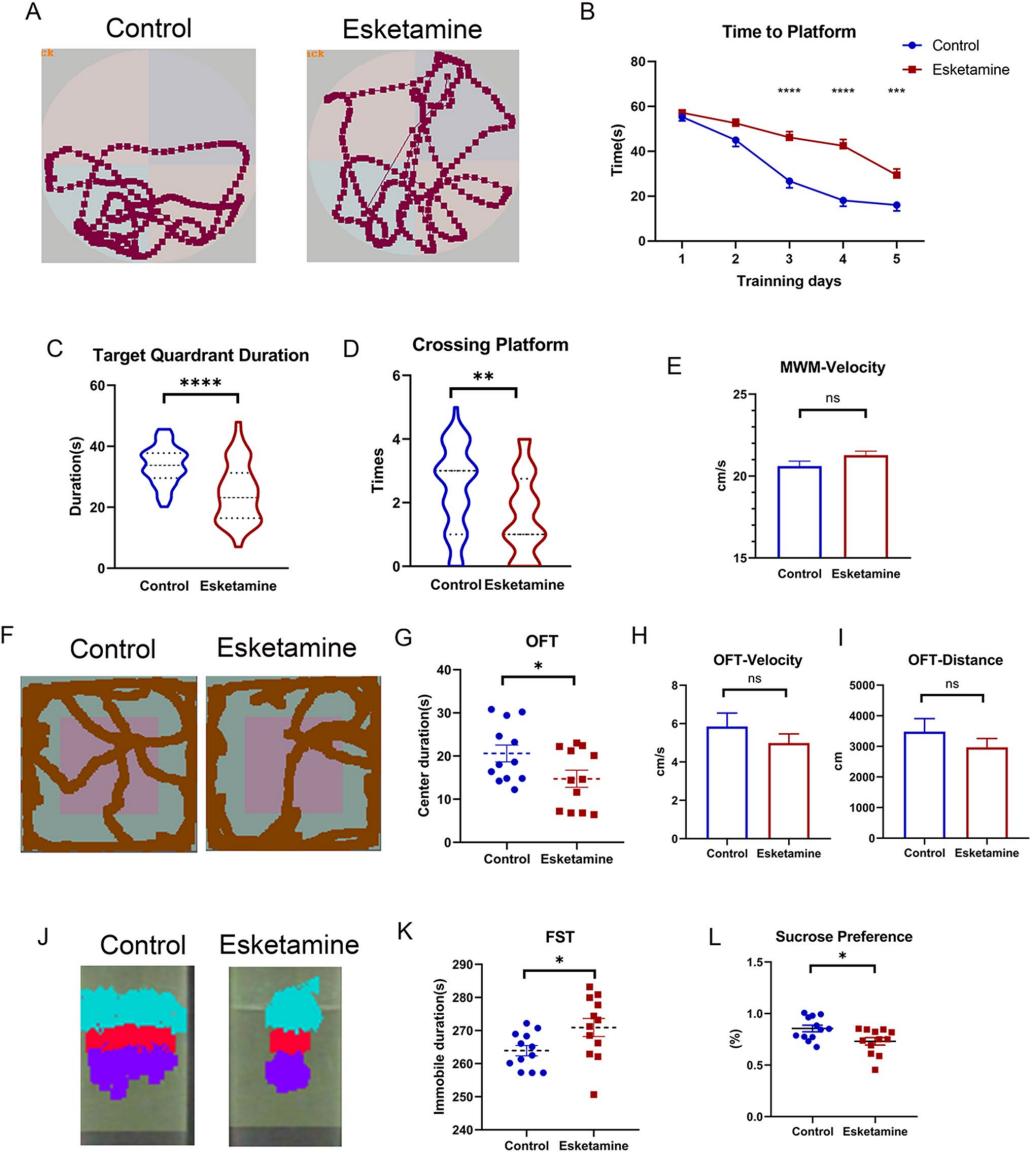
Impaired neuronal development induced behavioral deficiency. Twelve pups per group took part in each behavioral test (n = 12 pups/group). **A** is the representative diagram of the swimming trajectory of the rats, while **B** revealed the statistical analysis of time to platform during the five training days via ANOVA analysis. Time to platform were significantly longer in the esketamine group on day 3/4/5. The target quadrant duration was shorter (t test, 24.66 ± 1.39 s vs. 33.82 ± 0.89 s, P < 0.001) and the times crossing the platform was lesser (t test, 1.6 ± 0.2 vs. 2.4 ± 0.2, P = 0.002) on the test day in the esketamine group. Swimming velocity was similar between the two groups, showing adequate physical motor ability. **F** are the representative diagrams of the motoring trajectory in OFT. The traveling duration in the center zone of the pups was significantly shorter in the esketamine group (t test, 14.73 ± 1.98 s vs. 20.60 ± 1.95 s, P = 0.046), while the velocity and distance were similar. **J** Showed the performance of the rats in FST. Rats in the control group were struggling most of the time, while those in the esketamine group remained floating. Blue color stands for the motor fields for the head, red color for the body and purple color for the lower limbs of the rats. The immobile time was longer in the esketamine group (t test, 270.90 ± 2.72 s vs. 263.90 ± 1.56 s, P = 0.036) (**K**). Sucrose preference (**L**) was significantly lower in the esketamine group (0.73 ± 0.04 g vs. 0.86 ± 0.03 g, P = 0.017)

The hippocampus is crucial for cognition, memory and emotion (Anacker and Hen 2017). Therefore, we performed the following behavioral tests for spatial memory and mood disorder assessment. Twelve pups/group received 5 days of MWM training with a platform in the pool on P24-28 and were tested on P29 with platform withdrawn (Fig. 6A–E). On day 3/4/5, the mean time to platform of offspring rats in control group is 26.7 s/18.1 s/16.0 s, while that in esketamine group is 46.2 s/42.4 s/29.3 s, with remarkable significance (P < 0.0001, P < 0.0001, P < 0.001). Moreover, the target quadrant duration was shorter (t test, 24.7 ± 1.4 s vs. 33.8 ± 0.9 s, P < 0.001) and the number of platform crossings was lower (t test, 1.6 ± 0.2 vs. 2.4 ± 0.2, P = 0.002) on the test day in the esketamine group. The swimming velocity of rats was 20.61 cm/s in the control group and 21.28 cm/s in the esketamine group, indicating that the physical mobility was similar between the two groups. On P28, another 12 pups were tested in the open field (Fig. 6F–I). The traveling duration in the center zone of the pups was significantly shorter in the esketamine group (t test, 14.7 ± 2.0 s vs. 20.6 ± 2.0 s, P = 0.046). The velocity and travelled distances were not significantly different between the two groups, indicating similar physical mobility. After 3 h of rest, the pups were left in the swimming pool for 2 min of adaptation and 5 min of forced swimming test. Rats in the control group were struggling most of the time, while those in the esketamine group remained floating. The immobile time was longer in the esketamine group (t test, 270.9 ± 2.7 s vs. 263.9 ± 1.6 s, P = 0.036). On the evening of P28, pups were left in independent cages with one pure water bottle and one 1% sucrose bottle and food. Then, they were fasted for 8 h on the next day and given pure water and sucrose for a 1-h test. Sucrose preference was significantly lower in the esketamine group (0.73 ± 0.04 g vs. 0.86 ± 0.03 g, P = 0.017). Offspring rats in the esketamine group behaved more badly than the control group.

## Discussion

Our current study revealed that G14.5 esketamine administration influenced the neurobehavior of the offspring in adolescence. Poorer neuronal growth was revealed in the P0 hippocampal neuron culture in the esketamine group. Brain proliferative capacity in late gestation and juvenile pups was reduced in the esketamine group, resulting in impaired P30 neuronal plasticity and fewer synaptic spines. Attenuated LTP reflected compromised hippocampal function, as confirmed by behavioral tests of cognition and memory as well as emotional assessment.

Evidence from the clinical anesthesia indicated that esketamine is superior to ketamine, owing to the following reasons. First, the anesthetic effect of esketamine is twice as potent as racemic ketamine (Adams and Werner 1997; White et al. 1985). Second, due to the higher anesthetic effect, the lower dose needed reduces the dose-dependent side effects of the drug, such as hallucination (Bowdle et al. 1998) and cognitive impairment (Pfenninger et al. 2002). Third, the elimination of esketamine is faster than ketamine (Wang et al. 2019), which makes it more favorable for use in anesthesia as it can be better controlled (Adams and Werner 1997). Esketamine offers a shorter recovery time rather than ketamine (Wang et al. 2019; White et al. 1985).

To the best of our knowledge, there is no existing literature comparing the prenatal/perinatal use of esketamine with ketamine, neither in human nor animal research. However, previous researchers have focused on the prenatal use of ketamine on the neurodevelopment in offspring. Ketamine impaired the offspring's postnatal neurogenesis when administered in G14.5 pregnancy and it also led to neuronal apoptosis (Zhao et al. 2016, 2014), cognitive dysfunction and mood disorder via the BDNF/CREB pathway (Li et al. 2017b) and neurotoxic effect through Wnt/β-catenin pathway (Zhang et al. 2020). Cellular proliferative inhibition was dose-dependently related to prenatal ketamine exposure (Dong et al. 2016). Esketamine has a higher affinity for NMDA receptors than ketamine and requires a lower dose in clinical work, which may exert different effects on the offspring. Our results showed reduced brain proliferative capacity and neuronal growth in offspring, which is consistent with previous research on ketamine (Dong et al. 2012; Zhao et al. 2014). In addition, a decrease in spine density was found in the hippocampal region of the offspring, which is similar to the results of the ketamine study. The hippocampal NR1 level remained unchanged in ketamine researches (Ren et al. 2019; Zhao et al. 2014), the same as what we found in esketamine study. However, Zhao et al. found increased NR2A but decreased NR2B in the hippocampi of offspring with prenatal exposure to ketamine, and we found decreases in both NR2A and NR2B levels in offspring with prenatal exposure to esketamine. Similar neurobehavior was found in our esketamine study and other ketamine research, such as a longer escape latency in the MWM (Li et al. 2017a; Zhao et al. 2014) and less explorative performance in the OFT (Coronel-Oliveros and Pacheco-Calderon 2018) and increased immobile time in the FST (Coronel-Oliveros and Pacheco-Calderon 2018; Zhao et al. 2014) as well as decreased sucrose preference (Zhao et al. 2014).

The BDNF-CREB pathway has been widely acknowledged to be essential for memory and cognition (Cohen and Greenberg 2008; Egan et al. 2003; Hariri et al. 2003). Our results showed that the BDNF-CREB pathway in the hippocampi of the offspring was weakened in the esketamine group. BDNF, as an anterograde and retrograde neurotrophic factor, can be secreted from the cell body, dendrite, or axon in response to neuronal depolarization, and then be relieved from both axons and dendrites in response to excitatory synaptic activity (Benarroch 2015). BDNF promotes the survival and differentiation of neurons and induces neurite outgrowth during the development of the brain (Greenberg et al. 2009). As a self-amplifying autocrine signal, BDNF promotes axonal growth cone formation (Cheng et al. 2011; Yoshimura et al. 2005) and dendrite growth toward the adult pattern (Dijkhuizen and Ghosh 2005; Wirth et al. 2003), and triggers further secretion of BDNF (Cheng et al. 2011). Gestational administration of esketamine caused poor primary neuron growth in culture, with a shorter axonal length and fewer dendrite branches, which may be related to the decreased expression of BDNF. In addition, prenatal and postnatal proliferative capacity decreased, causing compromised neuronal plasticity in offspring rats on P30, with a lower number of newly emerging neuronal cells and mature neurons.

BDNF contributes to the activity-dependent increase in the number and volume of dendritic spines at glutamatergic synapses, which is essential for maintaining LTP (Jaworski et al. 2005; Tanaka et al. 2008). Our study showed that both morphology and function were influenced by the gestational administration of esketamine, with decreased spine density and attenuated LTP. The CREB family of transcription factors is the key mediator of the early phase of BDNF transcriptional autoregulation (Esvald et al. 2020). Previous studies showed that infusion of BDNF protein into the adult rat hippocampus results in transcription-dependent LTP via the phosphorylation of CREB (Messaoudi et al. 2002; Ying et al. 2002). CREB serine-133 phosphorylation is a reliable indicator for CREB activation and is involved in synaptic development, plasticity, and the pathogenesis of human disorders. Regulation of these activity-dependent genes has impact on cognitive development and function (Cohen and Greenberg 2008). Our results showed that the expression of phosphorylated CREB (p-CREB) was reduced in the esketamine group, which is consistent with the observed attenuation of long-term potentiation (LTP).

LTP is a persistent increase in synaptic strength, induced by repetitive activity during learning or experimentation (Alkadhi 2021). Synapses store information through continuous modification of their structure and molecular composition (Bosch et al. 2014). Synaptic transmission relies on the fast, efficient and synchronous release of chemical neurotransmitters in response to the action potential-dependent entry of extracellular calcium via voltage-gated channels (Cousin 2021), which is achieved with the help of mobile synaptic vesicles traveling to the postsynaptic membrane. Synaptophysin (SYP/SY38) is one of the most abundant cargos on synaptic vesicles (Cousin 2021), regulating at least two phases of endocytosis to ensure vesicle availability during and after sustained neuronal activity (Kwon and Chapman 2011). Postsynaptic density 95 is the key protein of the postsynaptic membrane, especially in the hippocampus (Ladurelle et al. 2012). Synaptic proteins play an important role in the synaptic formation, remodeling, and function, thus providing synaptic stability during brain development (Li et al. 2017b). Our results showed that the expression of SY38 and PSD95 were lower in P0 and P30 offspring in the esketamine group, indicating impaired synaptic stability and function. Synaptic dysfunction may account for clinical signs of dementia and cognitive decline through the disruption of neuronal communication (Coleman et al. 2004).

LTP involves an increase in the cytoplasmic free calcium ion (Ca^2+^) concentration in postsynaptic neurons (Alkadhi 2021), which is achieved through receptor channels such as the ionotropic glutamate receptor NMDAR. NMDAR interacts with many proteins of the postsynaptic density. Some of these proteins modulate glutamate receptor function, whereas others control their membrane insertion and removal. Therefore, the number of glutamate receptors at a synapse can be regulated through these interactions (Luscher and Malenka 2012). Hippocampal NMDARs can be diheteromeric (GluN1/GluN2A and GluN1/GluN2B) or triheteromeric (GluN1/GluN2A/GluN2B) (Shipton and Paulsen 2014), so we detected NR1, NR2A and NR2B in the hippocampi of P30 rats. We found that both NR2A and NR2B were expressed at lower levels in the esketamine group, while the NR1 level remained unchanged. A previous study showed that NR2A was increased in the hippocampi of offspring after prenatal exposure to ketamine, while NR2B decreased (Zhao et al. 2014), which is not completely consistent with our results. To some extent, both studies did find abnormalities in the NR2A/NR2B subunits, which may influence the induction of LTP. In addition, changes in the quantity or subcellular location of these subunits could alter the basal state of a spine, and thus affect the future induction of plasticity (Shipton and Paulsen 2014).

Overall, BDNF quantity and NMDAR-dependent synaptic neurotransmission inducing LTP via the CREB phosphorylation are the structural fundamental to hippocampal function and is linked to memory and mood regulation. In rodent animal experiments, the Morris water maze is the classic test measuring spatial cognition and memory ability, while the open field test, sucrose preference test and forced swimming test are usually used for anxiety and depression assessment (Bannerman et al. 2014). Gestational administration of the NMDAR antagonist esketamine impaired the formation and function of the hippocampus, leading to a behavioral deficit in the offspring rats. To our knowledge, this is the first study concentrating on the effect of perinatal use of the S(+) isomer esketamine on offspring brain development.

Due to there is no directly comparison between esketamine and ketamine in prenatal anesthesia use, we could not draw a conclusion about which one is superior. Generally speaking, esketamine has higher potency and requests lower dose, which may cause fewer adverse effects. The short-term benefits of esketamine over Ketamine during the perioperative period have been demonstrated in non-pregnant surgical patients. Hence, it is crucial to further investigate the potential long-term effects of esketamine, particularly in prenatal use, on offspring in future studies. Moreover, comparison of prenatal/perinatal use of ketamine and esketamine would be better for guiding the clinical decisions for pregnant patients.

There are several limitations of our study.

First, the esketamine dose we used on the pregnant rats was not equal to that used in clinical practice. According to the extrapolation principle between animals and humans (Reagan-Shaw et al. 2008), the esketamine dose in rats should be 3.1 mg/kg. However, clinical anesthesia work is usually achieved by multimodal anesthetic techniques with compound drugs. The single use of a clinical dose of esketamine is not able to fully sedate/anesthetize the rats. Therefore, considering that esketamine was twice as potent as ketamine (Domino 2010), and that the previously reported dose of ketamine in rats was 50 mg/kg intramuscularly (Green et al. 1981), whereas the previous dose of ketamine used in pregnant rats was 40 mg/kg intravenously (Zhao et al. 2014), we chose 20 mg/kg esketamine intravenously for our study in pregnant rats. An excessive dose would inevitably lead to drug toxicity. Second, the 2-h anesthetic time during a 21–23 day pregnancy in rats is equivalent to 24–26 h in a human pregnancy (280 days), which is generally not realistic in clinical work. Excessive anesthetic exposure time causes side effects, as shown in our results.

Real-world human research, such as cohort studies, is needed, and multicenter research is better. However, anesthetic exposure in humans is usually accompanied by surgeries to cure diseases. The effects of the primary disease and surgery stress cannot be ignored. In this case, animal study designs could avoid the surgery procedure, and we indeed followed this rule, although modification of the study design is needed in future studies, such as reducing the anesthetic time. The subject of our study, esketamine, is not only an anesthetic but also one of the antidepressants. Therefore, clinical cohort studies of this drug in psychiatry may add some evidence to its use in anesthesia, and we expect coordinated drug research between the two fields to shed more light on the effects of esketamine administered during pregnancy on the offspring.

In summary, our findings suggest that maternal exposure to esketamine affects neuronal development in offspring through decrease phosphorylation of CREB, leading to neurobehavioral deficits in rats.

## Supplementary Information

Below is the link to the electronic supplementary material.

Supplementary file1 (DOCX 11513 KB)

Supplementary file2 (DOCX 15 KB)

Supplementary file3 (DOCX 19 KB)

## Data Availability

The data that support the findings of this study are available from the corresponding author, Yalan Li, upon reasonable request.
