# Supplementary material for: Prenatal Exposure to General Anesthesia Drug Esketamine Impaired Neurobehavior in Offspring
Source: Cell Mol Neurobiol. 2023 Apr 29;43(6):3005–22. doi: 10.1007/s10571-023-01354-4 (PMC10333374; doi:10.1007/s10571-023-01354-4)
Supplement: Supplementary file 1 — Supplementary file1 (DOCX 11513 KB) [file 10571_2023_1354_MOESM1_ESM.docx]

**BDNF**

**BDNF images in Figure 5. (The red rectangle)**


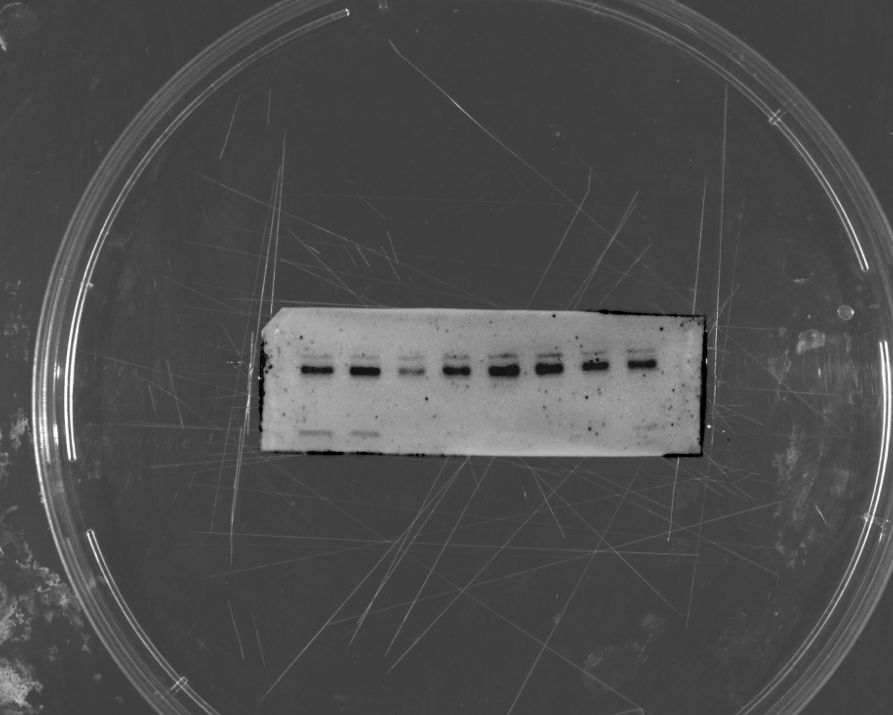


15kDa

10kDa

P0 Esketamine

P30 Esketamine

P30 Control

P0 Control

β Tubulin


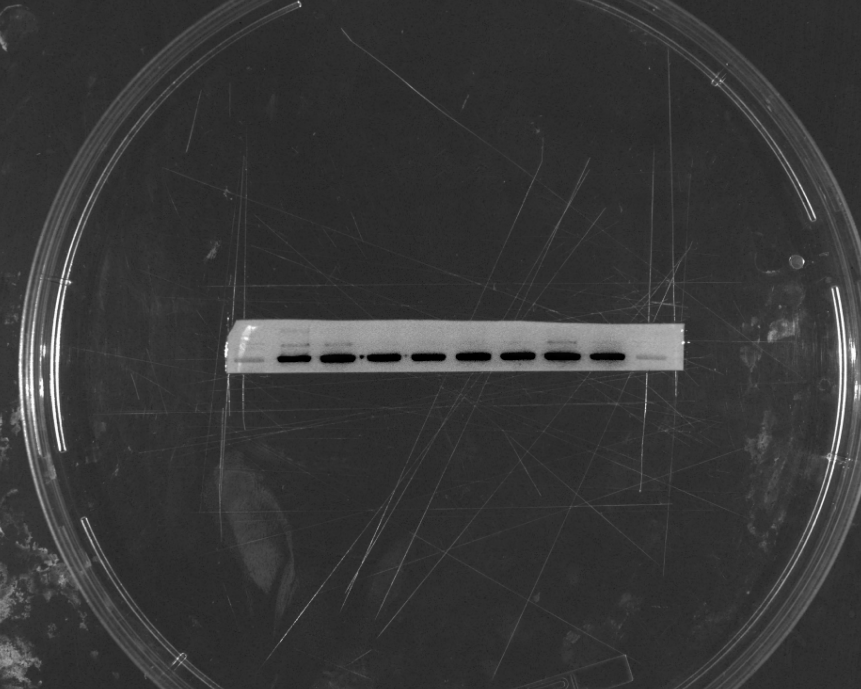


55kDa

P0 Esketamine

P30 Esketamine

P30 Control

P0 Control

**Replicates**

P0-BDNF

β Tubulin


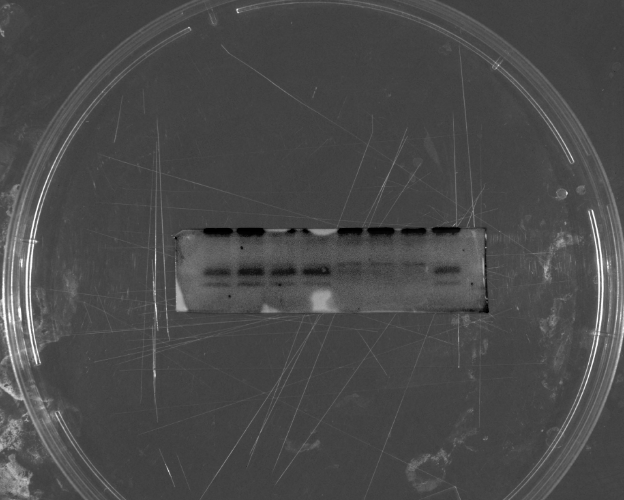

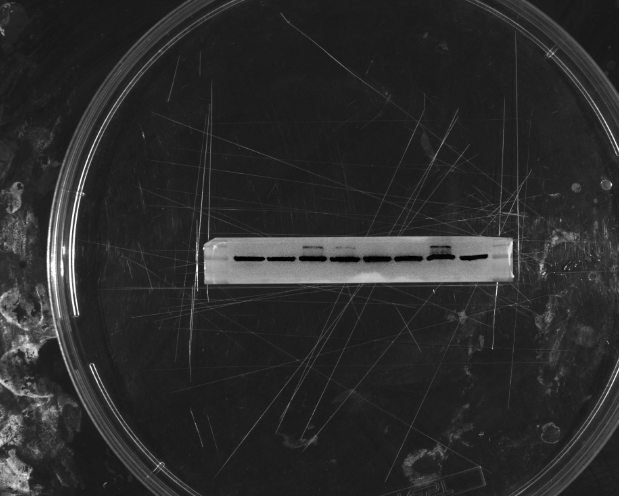


P0 Esketamine

P0 Control

P0 Esketamine

P0 Control

55kDa

15kDa

P30-BDNF

β Tubulin


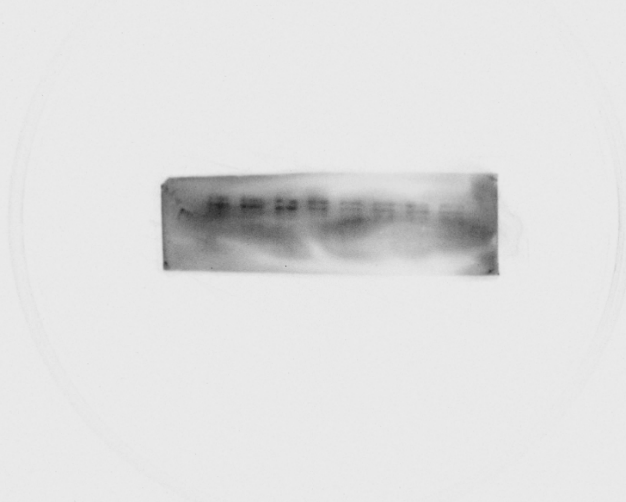

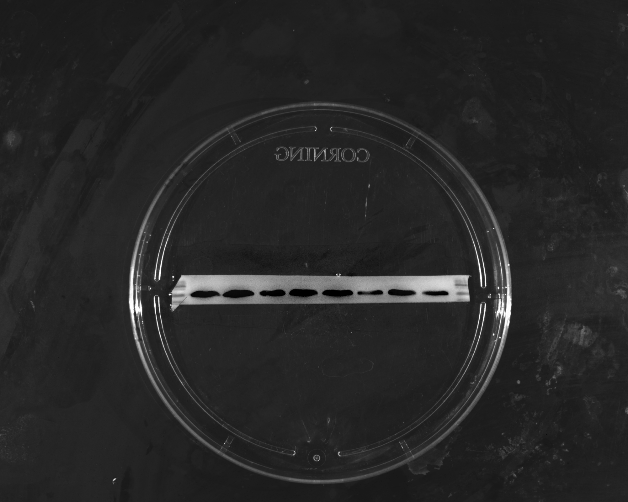


P30 Control

P30 Esketamine

P30 Esketamine

P30 Control

**SY38/Synaptophysin**

**SY38 images in Figure 5(The red rectangle)**


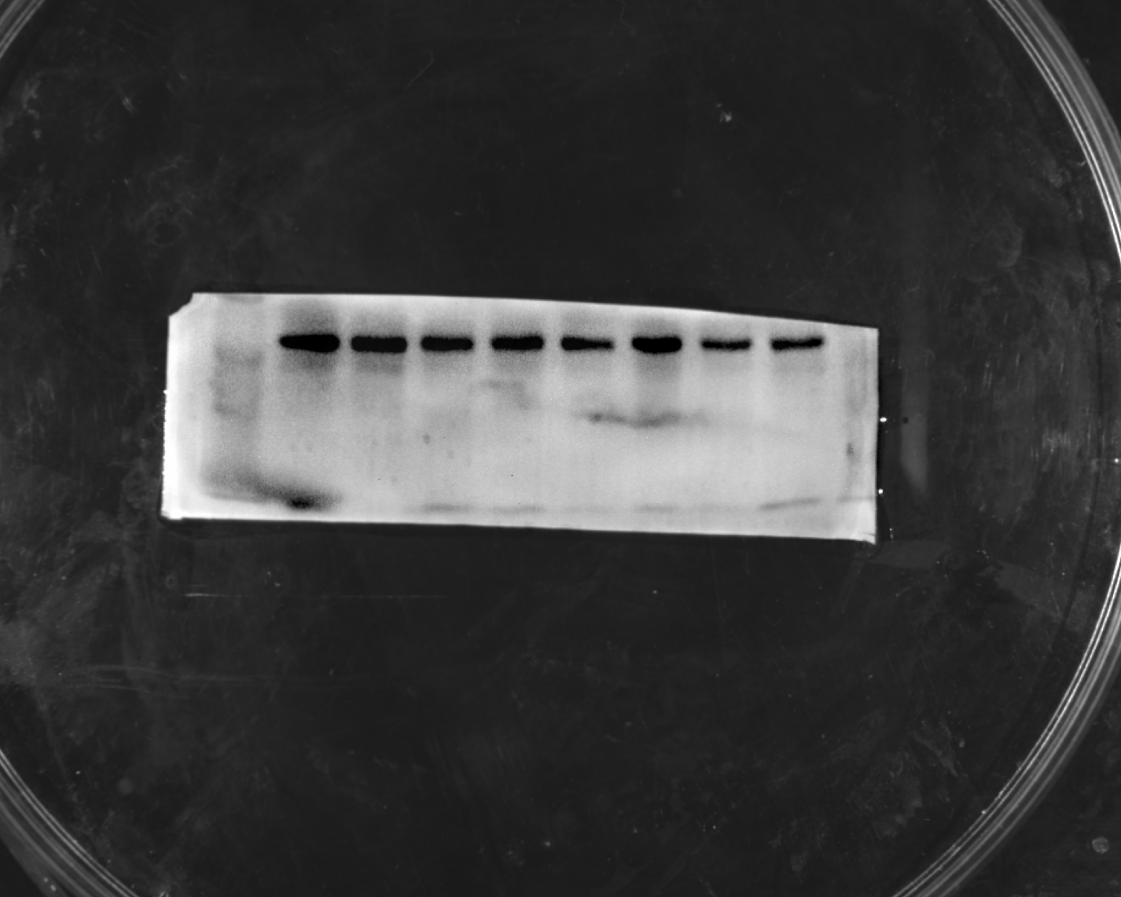


β Tubulin

SY38

25kDa

35kDa

40kDa

P30 Esketamine

P30 Control

P30 Esketamine

P30 Control

P0 Esketamine

P0 Esketamine

P0 Control

P0 Control


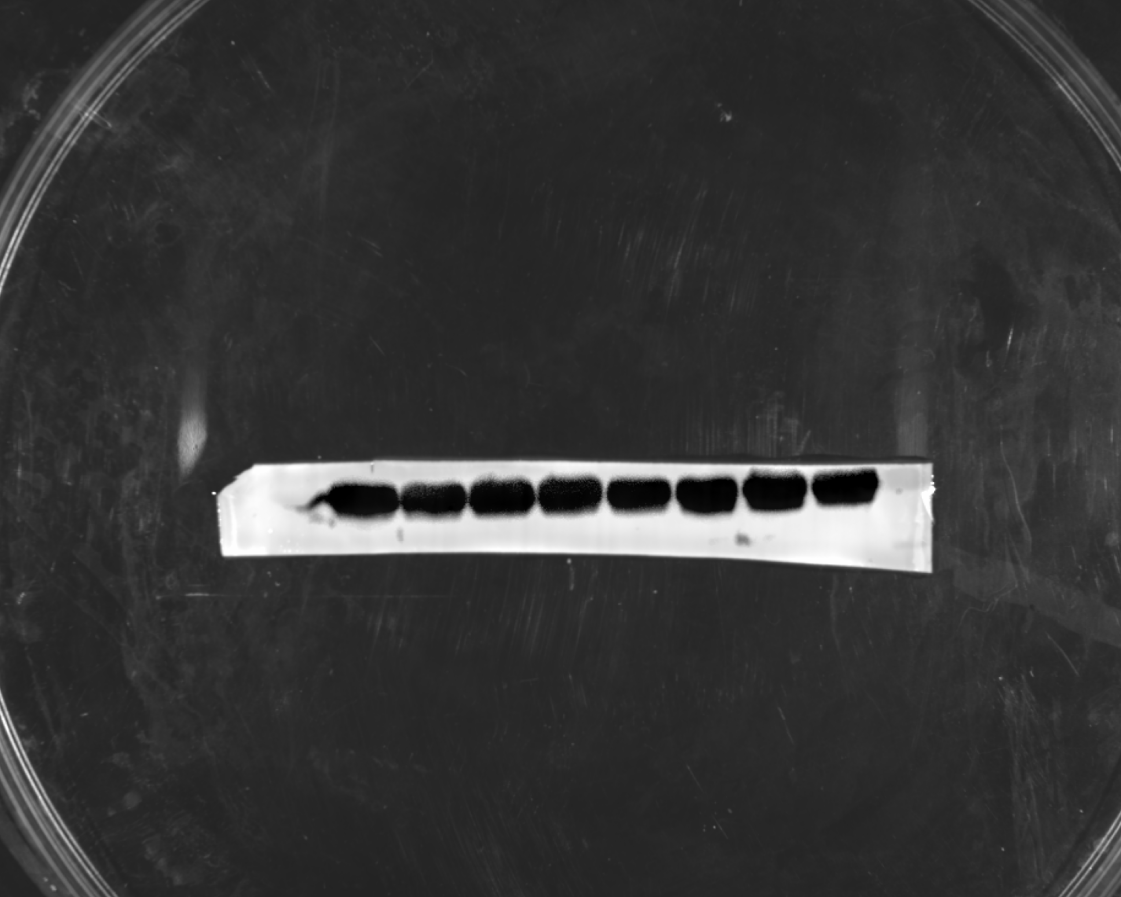


P30 Esketamine

P30 Esketamine

P30 Control

P30 Control

P0 Control

P0 Esketamine

P0 Esketamine

P0 Control

**Replicates**

M: marker; C: Control group; E: Esketamine group; X: blank.

Sample order: M C E M C E M C X M C E M C E

P0-SY38

β Tubulin


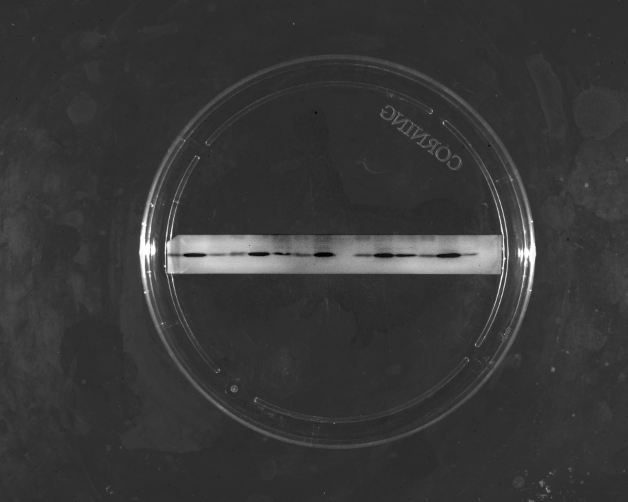

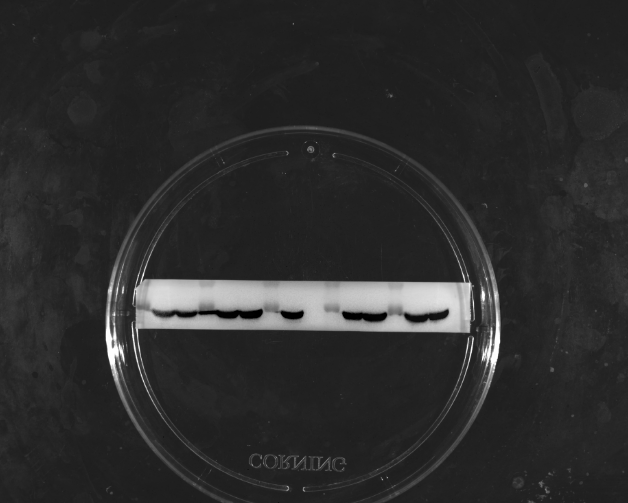


Blank

Blank

55kDa

35kDa

M: marker; C: Control group; E: Esketamine group.

Sample order: M C E M C E M C E M C E M C E

β Tubulin


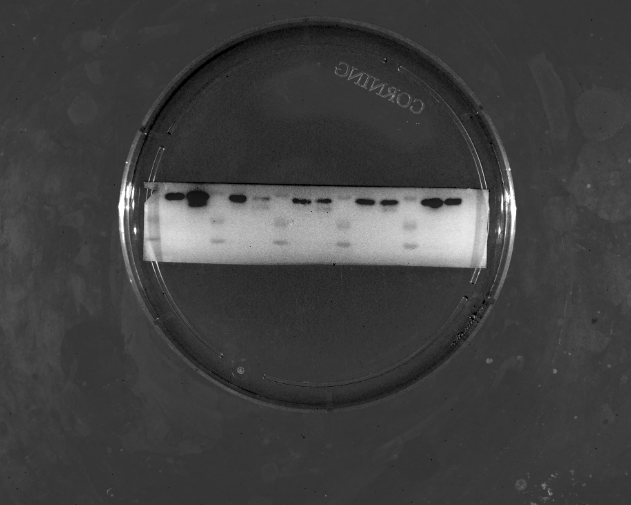

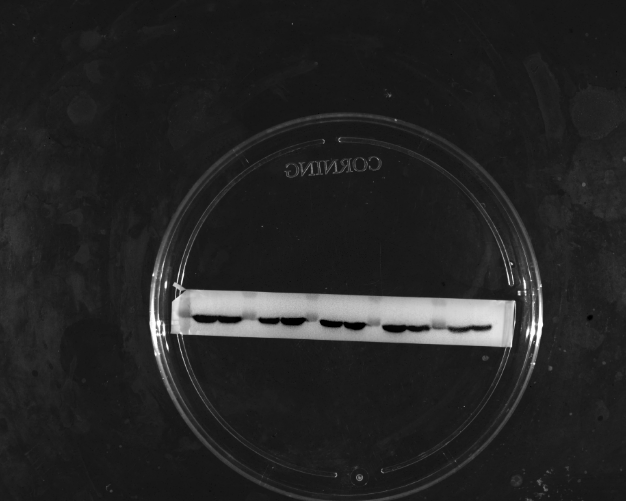


P30-SY38

55kDa

35kDa

**PSD95**

**PSD95 images in Figure 5(The red rectangle)**

P30 Esketamine


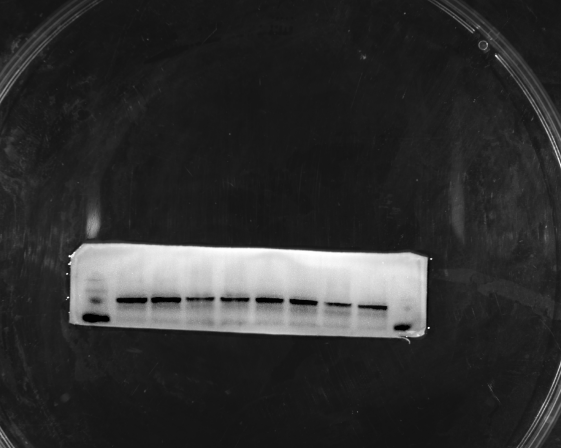

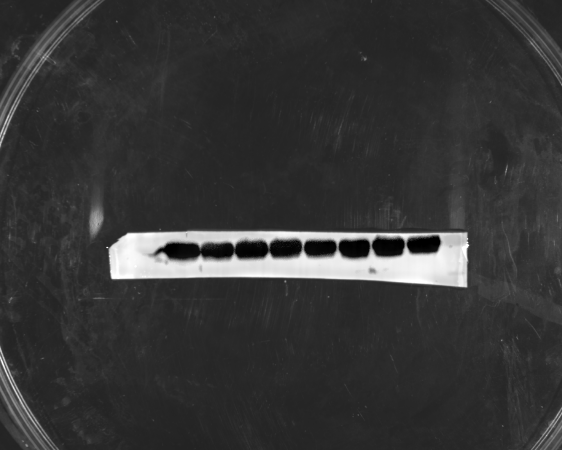


P30 Control

P0 Control

P0 Esketamine

P0 Esketamine

P30 Esketamine

P30 Control

P0 Controlc

PSD95

β Tubulin

70kDa

100kDa

**Replicates:**

M: marker; C: Control group; E: Esketamine group.

Sample order: M C E M C E M C E M C E M C E


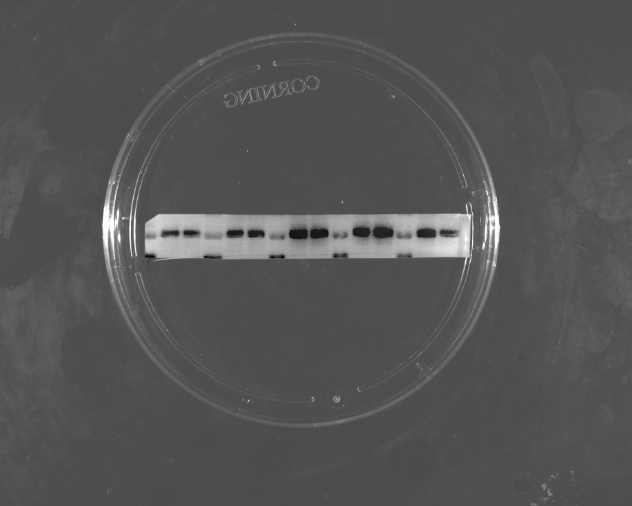

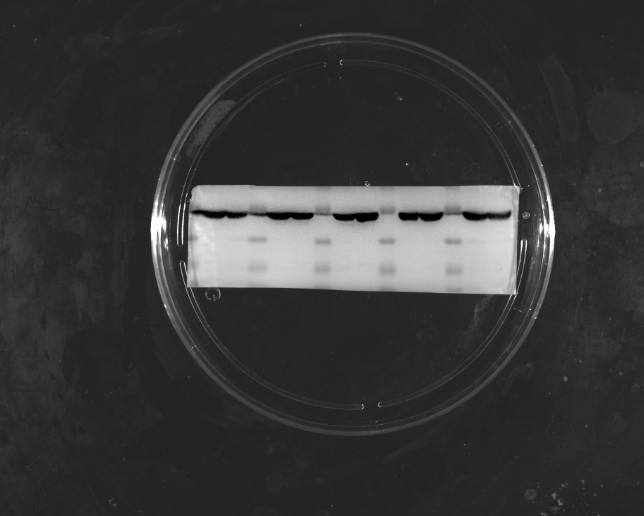


P0-PSD95

55kDa

β Tubulin

100kDa

70kDa


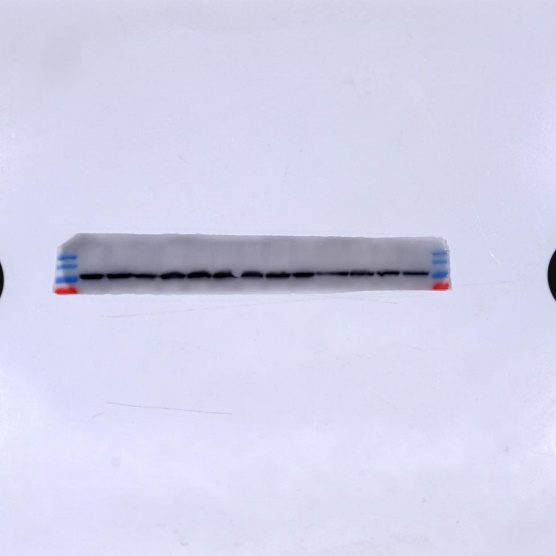

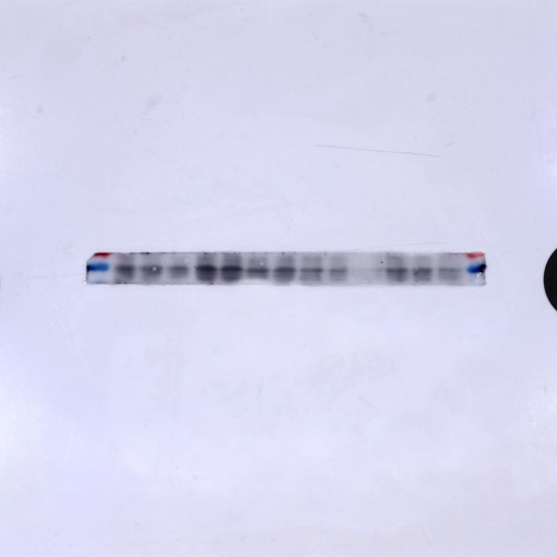


P30-PSD95

55kDa

P30 Esketamine

P30 Control

100kDa

P30 Control

P30 Esketamine

70kDa

β Tubulin

**p-CREB/CREB**

**p-CREB/CREB images in Figure 5(The red rectangle)**


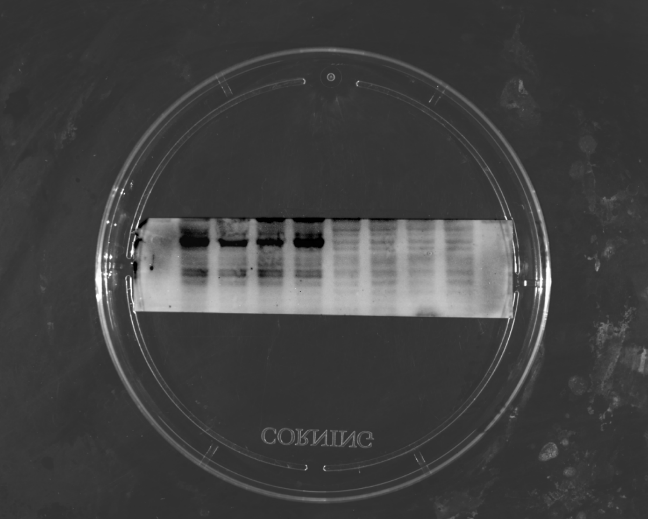

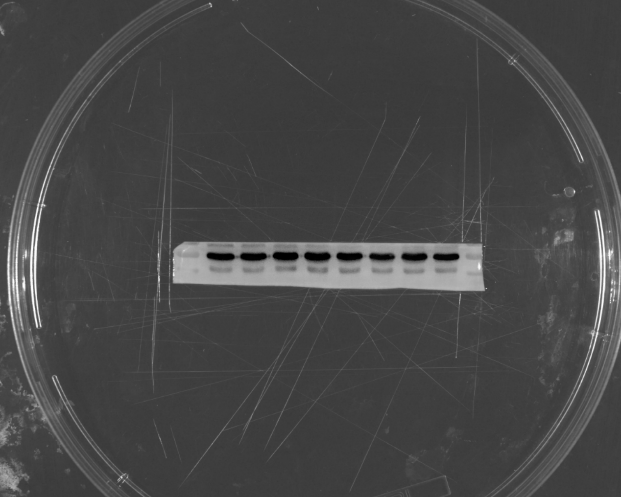


P0-p-CREB

CREB

40kDa

Other experiment samples

P0 Control

P0 Esketamine

Other experiment samples

P0 Esketamine

P0 Control

40kDa


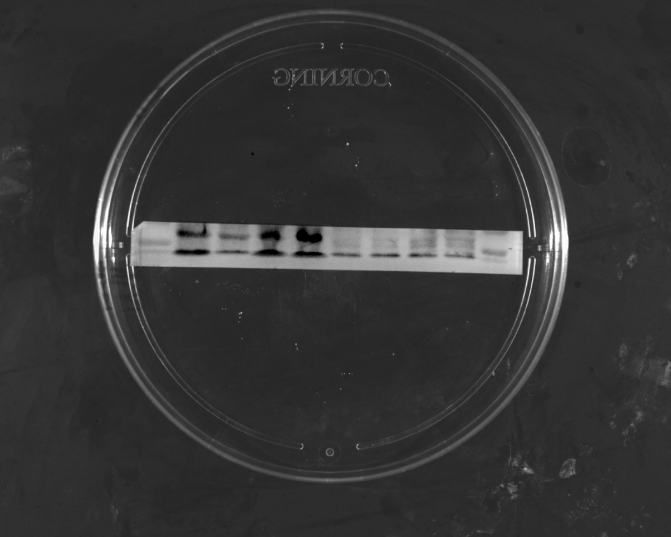

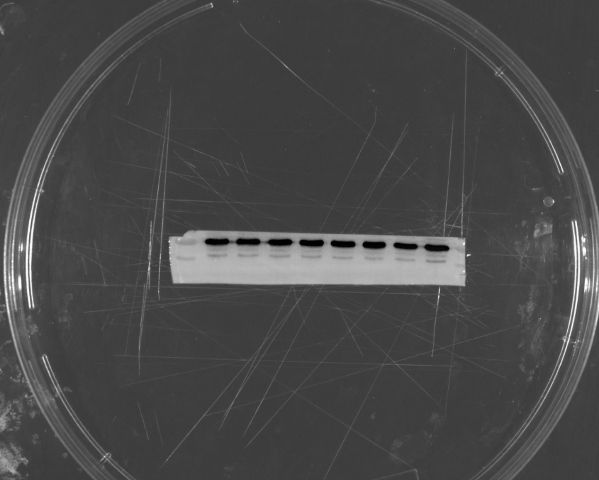


P30-p-CREB

CREB

Other experiment

samples

40kDa

P30 Esketamine

P30 Control

Other experiment

samples

P30 Control

P30 Esketamine

40kDa

**Replicates:**

M: marker; C: Control group; E: Esketamine group.

Sample order: M C E M C E M C E M C E M C E


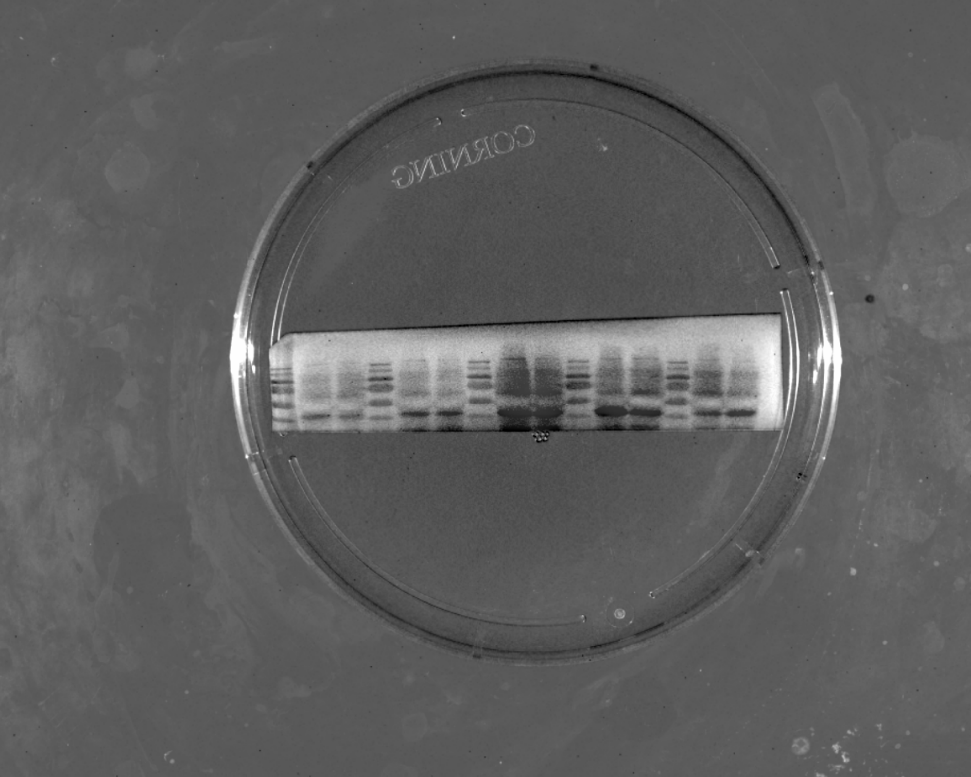

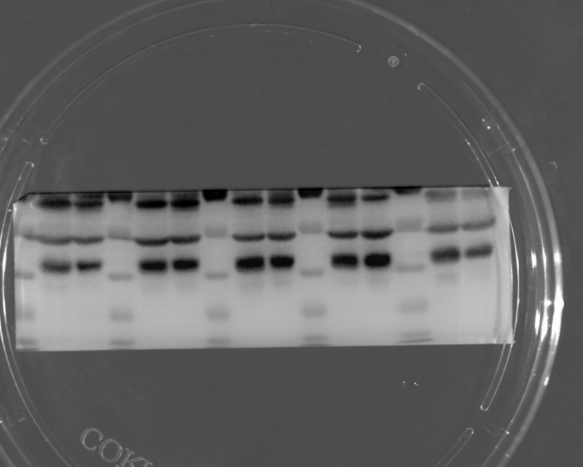


P0: CREB

P0: p-CREB

40kDa

40kDa


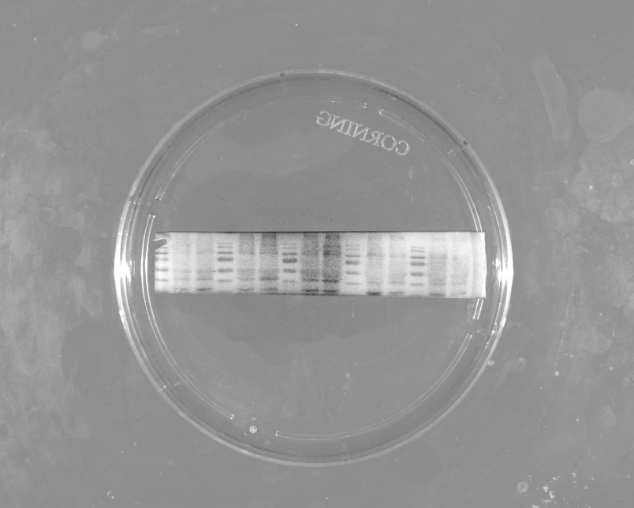

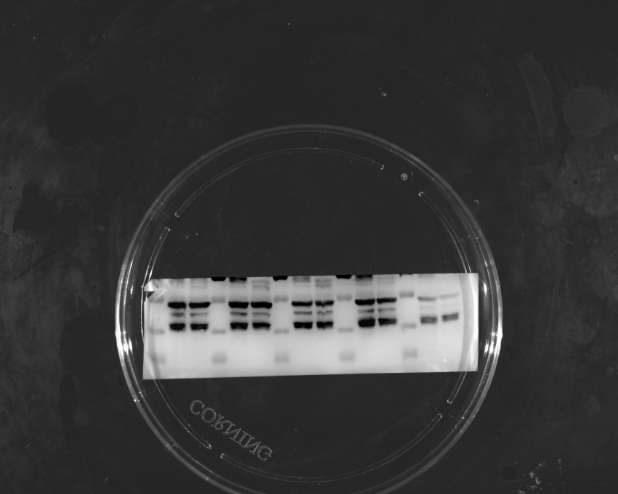


P30: CREB

P30: p-CREB

M: marker; C: Control group; E: Esketamine group.

**NR1**

**NR1 images in Figure 5(The red rectangles)**


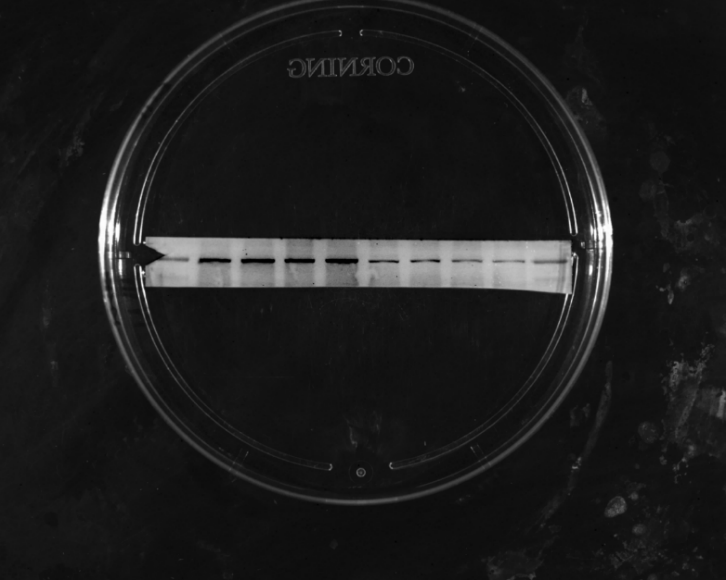


130kDa

P30 Esketamine

P30 Control

P30 Esketamine

P30 Control

Other experiment samples

Tubulin


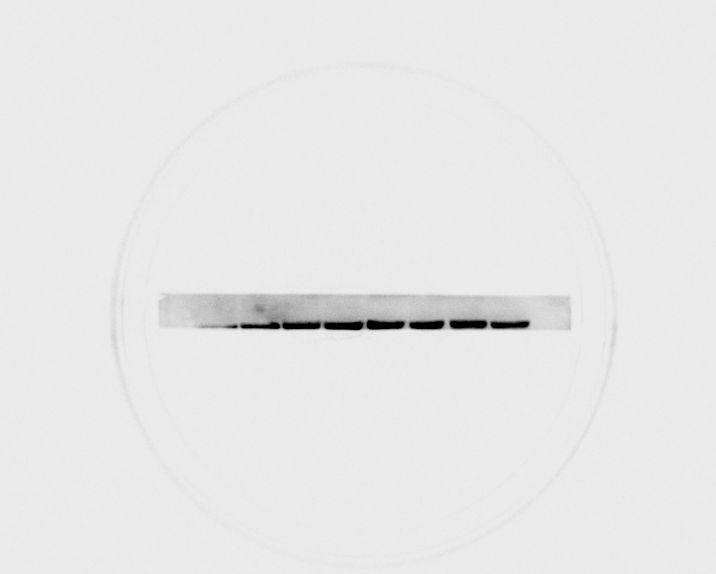


Other experiment samples

P30 Control

P30 Esketamine

P30 Esketamine

P30 Control

Replicates:

**P30: M C E M C E M C E M C E M C E**

M: marker; C: Control group; E: Esketamine group.


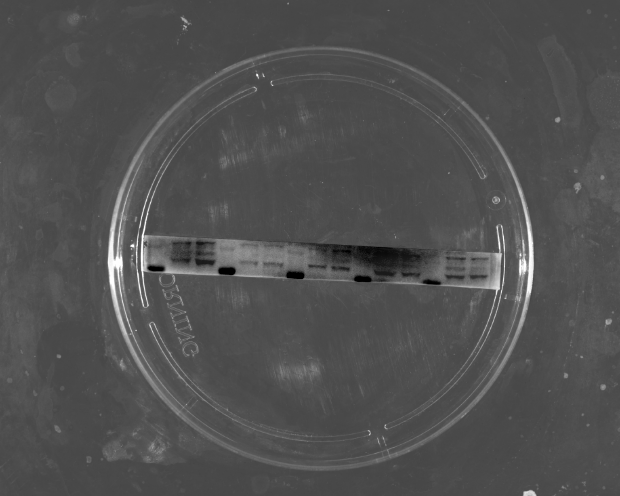

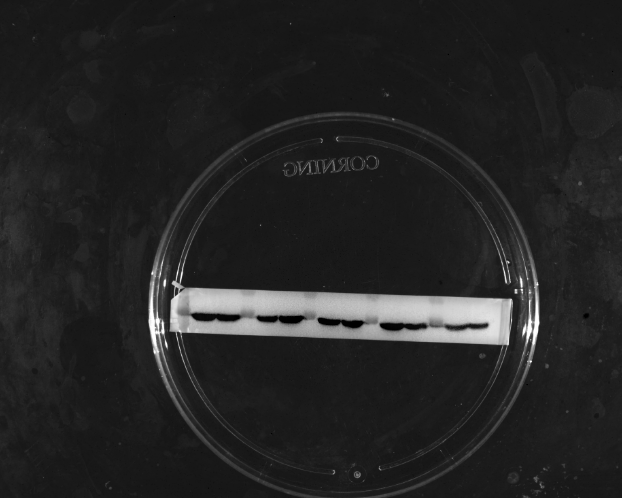


**NR2A**

**NR2A images in Figure 5(The red rectangles)**


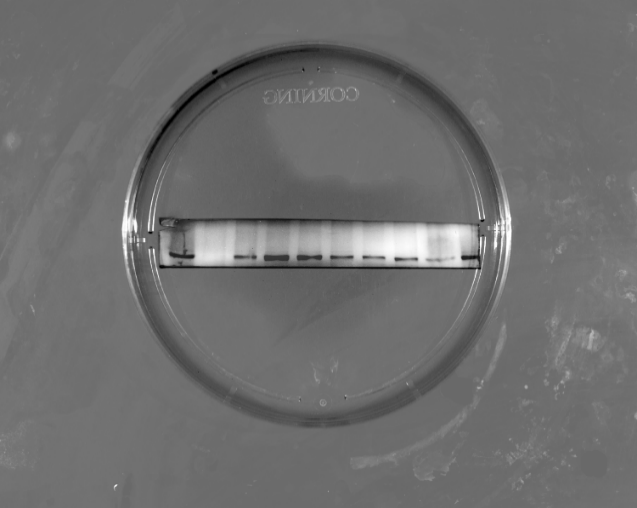

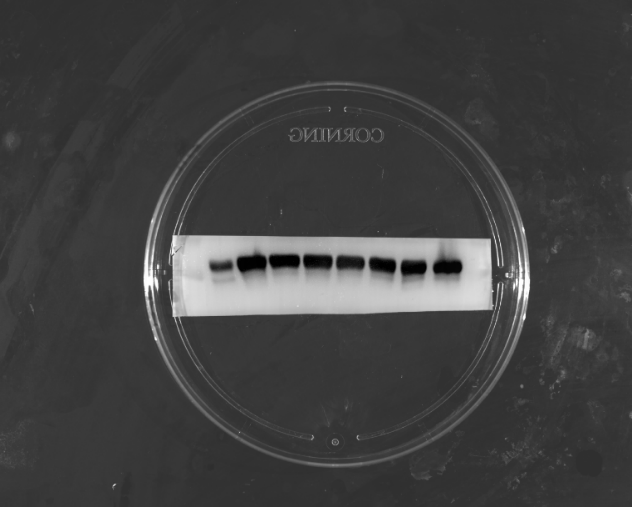


Proteolysis

Proteolysis

P30-NR2A

β Tubulin

55kDa

P30 Esketamine

P30 Control

170kDa

P30 Control

P30 Esketamine

**Replicates:**

**P30: M C E M C E M C E M C E M C E**


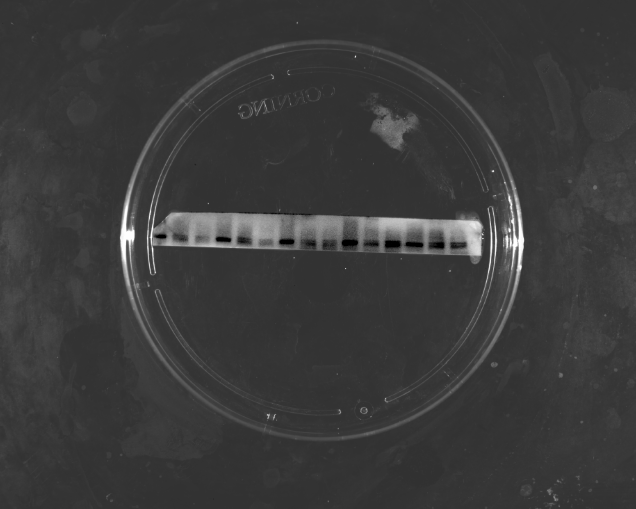

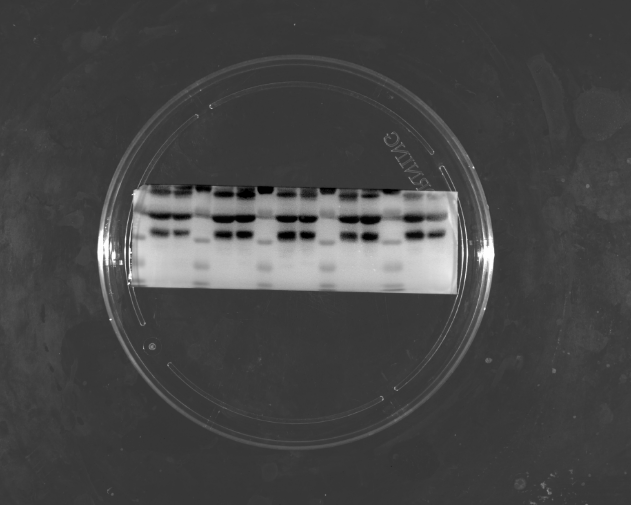


P30-NR2A

β Tubulin

M: marker; C: Control group; E: Esketamine group.

**NR2B**

**NR2B images in Figure 5(The red rectangles)**


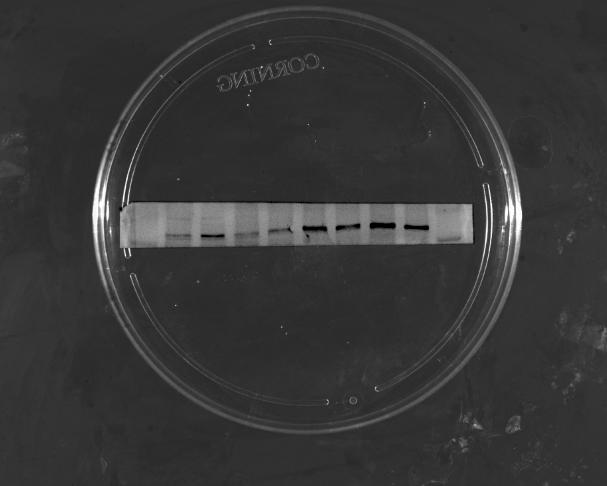

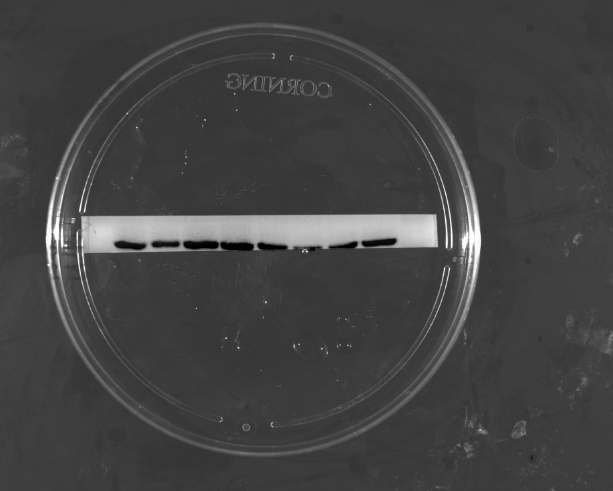


P30 Esketamine

P30 Control

P30-NR2B

Other experiment

samples

Other experiment

samples

P30 Esketamine

P30 Esketamine

P30 Control

P30 Control

β Tubulin

P30 Esketamine

P30 Control

**Replicates:**


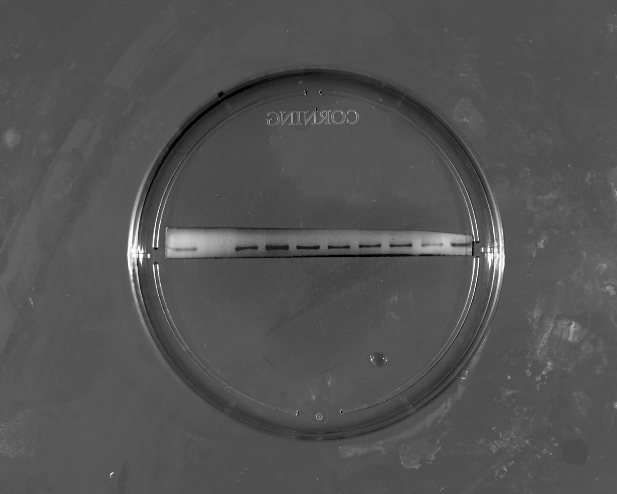

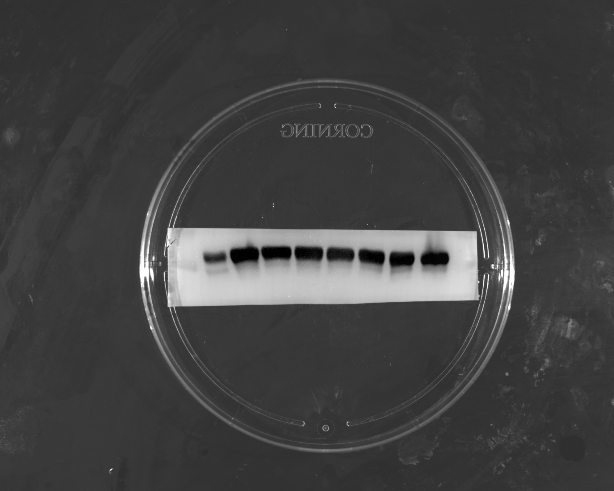


Proteolysis

Proteolysis

β Tubulin

P30-NR2B

170kDa

P30 Esketamine

P30 Control

P30 Esketamine

P30 Control
