# Supplementary material for: Prenatal Exposure to General Anesthesia Drug Esketamine Impaired Neurobehavior in Offspring
Source: Cell Mol Neurobiol. 2023 Apr 29;43(6):3005–22. doi: 10.1007/s10571-023-01354-4 (PMC10333374; doi:10.1007/s10571-023-01354-4)
Supplement: Supplementary file 2 — Supplementary file2 (DOCX 15 KB) [file 10571_2023_1354_MOESM2_ESM.docx]

**Abbreviations**

LTP: Long-term potential

SVZ: Subventricular zone

DG: Dentate gyrus

NMDAR: N-methyl-D-aspartic acid receptor

G: Gestational day.

P: Postnatal day.

EdU: 5-Ethynyl-2’-deoxyuridine.

MWM: Morris water maze test.

OFT: Open field test.

FST: Forced swimming test.

SPT: Sucrose preference test.

WB: Western blot.

qPCR: Quantitative real-time PCR.

IF: Immunofluorescence.

BDNF: Brain-derived neurotrophic factor

Sy38: Synaptophysin.

PSD95: Postsynaptic density 95.
