# Supplementary material for: Prenatal Exposure to General Anesthesia Drug Esketamine Impaired Neurobehavior in Offspring
Source: Cell Mol Neurobiol. 2023 Apr 29;43(6):3005–22. doi: 10.1007/s10571-023-01354-4 (PMC10333374; doi:10.1007/s10571-023-01354-4)
Supplement: Supplementary file 3 — Supplementary file3 (DOCX 19 KB) [file 10571_2023_1354_MOESM3_ESM.docx]

**Antibodies used in this study.**

| **Primary antibody** |  |  |  |  |
| --- | --- | --- | --- | --- |
| **Name** | **Company, developer** | **Catalog** | **Host** | **RRID** |
| NeuN | Abcam, USA | ab104224 | mouse | AB_10711040 |
| DCX | Abcam, USA | ab18723 | rabbit | AB_732011 |
| beta III tubulin | Abcam, USA | ab18207 | rabbit | AB_444319 |
| BDNF | Abcam, USA | ab108319 | rabbit | AB_10862052 |
| SY38 | Abcam, USA | ab8049 | mouse | AB_2198854 |
| PSD95 | CST, USA | 3450 | rabbit | AB_2292883 |
| p-CREB | CST, USA | 9198 | rabbit | AB_2561044 |
| CREB | PTM, China | 5595 | mouse |  |
| β-Tubulin | CST, USA | 2146 | rabbit | AB_2210545 |
| NR1 | Millipore, USA | 05-432 | mouse | AB_10015247 |
| NR2A | Millipore, USA | 07-632 | rabbit | AB_310837 |
| NR2B | Millipore, USA | 06-600 | rabbit | AB_310193 |
| **Secondary antibody** |  |  |  |  |
| Donkey anti-mouse 488 IgGs | Thermofisher, USA | A21202 | donkey | AB_141607 |
| Donkey anti-rabbit 546 IgG | Thermofisher, USA | A10040 | donkey | AB_2534016 |
| HRP Goat anti-rabbit | Abcam, USA | ab6721 | goat | AB_955447 |
| HRP Goat anti-mouse | Abcam, USA | ab6789 | goat | AB_955439 |
| DAPI | CST, USA | 4083 |  |  |

CST: Cell Signaling Technology, USA.

PTM: PTM Bio, Zhejiang, China.
